# *Pseudomonas aeruginosa N*-3-Oxo-Dodecanoyl-Homoserine Lactone Impacts Mitochondrial Networks Morphology, Energetics, and Proteome in Host Cells

**DOI:** 10.3389/fmicb.2020.01069

**Published:** 2020-05-25

**Authors:** Henrik Josephson, Maria Ntzouni, Camilla Skoglund, Stig Linder, Maria V. Turkina, Elena Vikström

**Affiliations:** ^1^Department of Biomedical and Clinical Sciences, Faculty of Medicine and Health Sciences, Linköping University, Linköping, Sweden; ^2^Core Facility, Faculty of Medicine and Health Sciences, Linköping University, Linköping, Sweden

**Keywords:** bacteria–host interactions, quorum sensing, *N*-acylhomoserine lactone, *Pseudomonas aeruginosa*, mitochondria, mitochondrial dynamics, proteomics

## Abstract

Mitochondria play crucial roles in cellular metabolism, signaling, longevity, and immune defense. Recent evidences have revealed that the host microbiota, including bacterial pathogens, impact mitochondrial behaviors and activities in the host. The pathogenicity of *Pseudomonas aeruginosa* requires quorum sensing (QS) cell–cell communication allowing the bacteria to sense population density and collectively control biofilm development, virulence traits, adaptation and interactions with the host. QS molecules, like *N*-3-oxo-dodecanoyl-L-homoserine lactone (3O-C_12_-HSL), can also modulate the behavior of host cells, e.g., epithelial barrier properties and innate immune responses. Here, in two types of cells, fibroblasts and intestinal epithelial cells, we investigated whether and how *P. aeruginosa* 3O-C_12_-HSL impacts the morphology of mitochondrial networks and their energetic characteristics, using high-resolution transmission electron microscopy, fluorescence live-cell imaging, assay for mitochondrial bioenergetics, and quantitative mass spectrometry for mitoproteomics and bioinformatics. We found that 3O-C_12_-HSL induced fragmentation of mitochondria, disruption of cristae and inner membrane ultrastructure, altered major characteristics of respiration and energetics, and decreased mitochondrial membrane potential, and that there are distinct cell-type specific details of these effects. Moreover, this was mechanistically accompanied by differential expression of both common and cell-type specific arrays of components in the mitochondrial proteome involved in their structural organization, electron transport chain complexes and response to stress. We suggest that this effect of 3O-C_12_-HSL on mitochondria may represent one of the events in the interaction between *P. aeruginosa* and host mitochondria and may have an impact on the pathogens strategy to hijack host cell activities to support their own survival and spreading.

## Introduction

*Pseudomonas aeruginosa* is an opportunistic Gram-negative pathogen that causes acute and chronic infections, mostly in patients with compromised health conditions. Being very flexible genetically, adaptable to different environments, resistant to multiple drugs and toxigenic, these bacteria can inhabit the host as an invasive pathogen or in the form of biofilms. *P. aeruginosa* harbors a sophisticated small molecule-based communication system, quorum sensing (QS), that allows the bacteria sense each other within population and collectively regulate the production of biofilms and virulent traits. Communication via QS gives microorganisms an advantage to behave as a coordinated, powerful multicellular community and enhance their pathogenicity and survival (Papenfort and Bassler, 2016; Turkina and Vikstrom, 2019). In *P. aeruginosa*, AHL-dependent QS network produces and detects two AHL signals: C_4_-HSL and 3O-C_12_-HSL. The AHL circuits control the activation of more than 300 genes in the bacterial genome including those that regulate iron metabolism, secretion systems, production of extracellular virulence factors, like elastases, proteases, exotoxin A, secretion system, and also exopolysaccharides and rhamnolipids important for biofilm development (Schuster and Greenberg, 2006; Irie and Parsek, 2008).

Bacteria have evolved a multitude of survival strategies adapted to their specific lifestyles, either extracellularly in biofilms or intracellularly in diverse mammalian cell types, thereby directly or indirectly interacting with intracellular compartments and organelles (Kim et al., 2010). Host organelles represent attractive targets for bacterial pathogens to hijack host cell activity and defense and secure their own survival and spreading. This targeting goes via mechanisms requiring bacterial QS communication and virulence factors such as toxins, enzymes and secretion system (Heimer et al., 2013; Garai et al., 2019).

Mitochondria are essential organelles forming dynamic networks within the eukaryotic cell. They resemble α-proteobacteria, with which they have close homology and from which they have evolved by endocytosis into a symbiotic relationship with eukaryotic cells (Han et al., 2019). Mitochondria have their own protein translation machinery and a separate small genome with a DNA code that is distinct from their bacterial ancestors and eukaryotic cell. The outer membrane separates them from the cytoplasm and surrounds an inner membrane, which is differentiated in the inner leaflet membrane and cristae that extend deeply into the matrix. These three compartments have distinct protein components and defined roles. Together, they drive cellular respiration, energy metabolism with the main source as ATP, production of NADH, guanine nucleotides, heme groups, and lipids. In parallel, they also affect cellular signaling pathways such as innate immunity, calcium signaling and stress responses. Conversion of bioenergy in mitochondria is performed by membrane protein complexes I–V, where ETC complexes and ATP synthase work together in oxidative phosphorylation to provide energy for the cell (Kuhlbrandt, 2015). Numerous mitochondrial functions and homeostasis are tightly linked to their dynamics, which is shaped by constantly ongoing fusion and fission events within their tubular network (Lackner, 2014). Hereby, they play a crucial role in cell homeostasis and this make them attractive targets for pathogens to interfere with their functions in order to maintain replicative niche or to support dissemination (Rudel et al., 2010; Ashida et al., 2011).

We and other researchers have previously explored how bacterial 3O-C_12_-HSL signals modulate host cell functions and responses by affecting various signaling pathways and perturbing different intracellular compartments, and these findings were recently reviewed (Turkina and Vikstrom, 2019). It has also been demonstrated that 3O-C_12_-HSL influences mitochondrial physiology and morphology by affecting ETC and releasing mitochondrial cytochrome c by activating caspases and without the involvement of Bak/Bax regulators of apoptosis (Schwarzer et al., 2014; Maurice et al., 2019).

The aim of this work was to address in a greater detail the consequences of bacteria–host cell communication at organelle and molecular levels, focusing on how 3O-C_12_-HSL impacts mitochondrial dynamics, architecture, respiration and energetic characteristics, and mitochondrial proteome. To achieve this, we used two types of mammalian cells, fibroblasts and intestinal epithelial cells, high resolution TEM, fluorescence imaging of living cells, mitochondrial respiration and energetics assays, quantitative mass spectrometry for mitoproteomics and bioinformatics. Indeed, we identified an array of multiple mitochondrial proteins being differentially expressed in response to 3O-C_12_-HSL and accompanied by a distinct effect on mitochondrial ultrastructure, dynamics, and energetic potential.

## Materials and Methods

### Cell Culture

Human epithelial colorectal adenocarcinoma Caco-2 cells (86010202 obtained directly from Sigma Aldrich, St. Louis, MO, United States) were grown in Dulbecco’s modified Eagle’s medium (DMEM) supplemented with 10% heat-inactivated fetal calf serum, 100 U/ml penicillin, 100 μg/ml streptomycin, 1% non-essential amino acids and 2 mM L-glutamine (Life Technologies, Grand Island, NY, United States) at 37°C in 5% CO_2_. This was done for 10–14 days to allow the Caco-2 cells to become mature and differentiated and to establish polarized epithelial monolayers. Mouse embryonal C3H10T1/2 fibroblasts (ATCC, Manassas, VA, United States) were cultured in DMEM supplemented with 10% heat-inactivated fetal calf serum, 100 U/ml penicillin and 100 μg/ml streptomycin (Life Technologies) at 37°C in 5% CO_2_. Fibroblasts were seed 1 day before experiments and grown overnight at 37°C in 5% CO_2_.

### AHL Synthesis

*N*-3-oxo-dodecanoyl-L-homoserine lactone C_16_H_27_NO_4_, MW 297.4 was synthesized by Prof. Peter Konradsson and Lan Bui (Department of Organic Chemistry, Linköping University, Sweden) as previously described (Chhabra et al., 2003). These molecules are structurally and functionally identical to those obtained from *P. aeruginosa* cultures. The resulting 3O-C_12_-HSL was checked for identity and purity by HPLC, and its activity as a QS-molecule was confirmed by the bioassays described earlier (Surette and Bassler, 1998; Winson et al., 1998).

### Treatment With AHL

For experiments, 3O-C_12_-HSL, dissolved in 100% dimethylsulfoxide (DMSO) as a stock solution, was first diluted in PBS, pH 7.3, and further in fresh medium to the desired final concentration. This mixture was used to replace the culture medium on cells. Thus, cells were treated with 10 or 50 μM 3O-C_12_-HSL for 1 or 3 h at 37°C in 5% CO_2_ and further proceeded for sample preparation and TEM. For Seahorse and proteome experiments, cells were induced with 10 or 50 μM 3O-C_12_-HSL for 3 or 4 h at 37°C in 5% CO_2_ and further proceeded. For real-time imaging, cells were treated with 3, 10, 50, or 100 μM 3O-C_12_-HSL. As vehicle for 3O-C_12_-HSL, 0.02% DMSO was used.

### Transmission Electron Microscopy

Cells grown on glass coverslips (thickness 0.17; Karl Hecht Assistent, Sondheim, Germany) in 6-well plates were fixed in 2% glutaraldehyde (Polyscience, Inc, Germany) in 0.1M Na cacodylate buffer, pH 7.4 at RT. The fixed samples were washed with the same buffer and post-fixed in 1% osmium tetroxide for 1 h at 4°C. Following block staining with 2% uranyl acetate in 50% ethanol, the samples were dehydrated in a series of ascending concentration of ethanol and acetone. A two-step infiltration was performed prior to embedding in Durcupan ACM epoxy resin kit (Sigma-Aldrich). The blocks were initially trimmed and sectioned using a Leica UC7 ultra microtome (Leica Microsystems GmbH, Vienna, Austria). Ultrathin sections of 60-nm thickness were collected onto formvar-coated copper slot grids, and counter-stained with uranyl acetate and lead citrate. TEM allowed capture and study subcellular architecture of the specimens at the micro- and nanoscale resolution. C3H10T1/2 fibroblasts specimens were examined in a JEM 1230 TEM operated at 100 kV (JEOL, Ltd, Tokyo, Japan); the images were taken with a Gatan Orius SC1000 CCD camera using Digital Micrograph software (Gatan, Pleasanton, CA, United States). Epithelial Caco-2 specimens were examined, and images were captured in a FEI Tecnai G2 (FEI Company, Hillsboro, OR, United States) at 200 kV and equipped with a Gatan US 4000 CCD camera (Gatan) and Tecnai Imaging and Analysis software (FEI Company). For further quantification, the images of the cells were analyzed using the ImageJ software (NIH, Bethesda, MD, United States). At least three independent experiments were done on separate days on different cell passages.

### Mitochondrial Respiration and Energetic Functions in Living Cells

**FIGURE 1:**
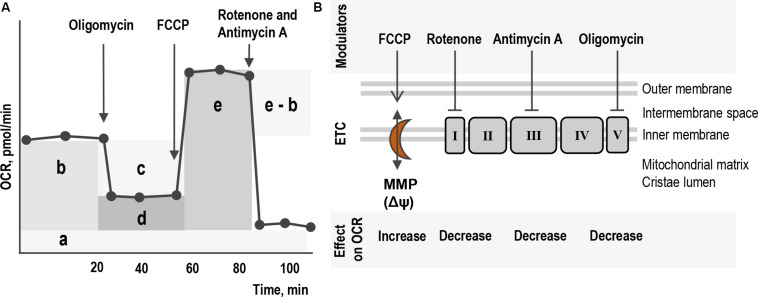
Schematic layout of the assay for mitochondrial respiration and energetics of living cells. **(A)** The profile of the major parameters of mitochondrial respiration in Seahorse Cell Mito Stress assay: non-mitochondrial respiration (a), basal respiration (b), ATP-linked respiration (c), proton leak (d), coupling efficiency (ratio between c and b), maximal respiration (e) and spare respiratory capacity (the difference between e and b). Mitochondrial respiration is measured as the oxygen consumption rate (OCR) of living cells over time in response to serial addition of modulators that target different components of the ETC in the mitochondria. **(B)** The four modulators targeting different components of ETC in mitochondria inner membrane in the cristae and their effect on the level of OCR. Oligomycin, inhibitor of complex V ATP synthase. Rotenone and Antimycin A, inhibitors of complexes I and III, respectively. FCCP, protonophore uncoupler targeting MMP. Five protein complexes I–V build together ETC and are involved in the process of oxygen-dependent oxidation and ATP production. Proton pumping across the cristae membrane is generated by three complexes: the NADH-ubiquinone oxidoreductase or complex I with NADH as the substrate produced in the citric acid cycle in the mitochondrial matrix; the ubiquinone-cytochrome c oxidoreductase or complex III; and the cytochrome c oxidase or complex IV with succinic acid as the substrate. Succinate dehydrogenase or complex II does not pump protons but contributes with reduced ubiquinone. The electron flow generates the difference in electron charge across the inner membrane, MMP. The gradient provides energy for ATP synthase or complex V to produce ATP.

The Seahorse XF24 Analyzer and Cell Mito Stress test kit 103015-100 (Agilent Technologies, Wilmington, DE, United States) were used to study major characteristics of respiration and mitochondrial function as shown in Figure 1A: non-mitochondrial respiration (a), basal respiration (b), ATP-linked respiration (c), proton leak (d), coupling efficiency (ratio between c and b), maximal respiration (e) and spare respiratory capacity (the difference between e and b). This was done by measuring the OCR of living cells over time in response to modulators that target different components of the ETC in the mitochondria (Figure 1B). These compounds, 1 μM oligomycin (inhibitor of complex V ATP synthase); 1 μM FCCP (protonophore uncoupler targeting MMP) and a mix of 0.5 μM rotenone and antimycin A (inhibitors of complexes I and III respectively), were serially added to the cells while measuring OCR (Figure 1A). All procedures for Cell Mito Stress experiments were assayed according to the manufacturer’s recommendations^1^. At least three independent experiments were done on separate days on different cell passages.

### Real-Time Imaging of Mitochondrial Membrane Potential

The MMP of living cells was assayed using the lipophilic cationic sensor JC-1 (Life Technologies) according to the manufacturer’s recommendations. Cells grown in 24-well plates were rinsed with DMEM without phenol red supplemented with 10% heat-inactivated fetal calf serum, 100 U/ml penicillin and 100 μg/ml streptomycin (Life Technologies) and stained with 5 mg/ml JC-1 for 45 min at 37°C in 5% CO_2_. Dye solution was then removed, and cells were washed twice in the same media before imaging. Green and red fluorescent images and bright field images of cells were acquired using IncuCyte Zoom live cell imaging and analysis system (Essen BioScience, Ann Arbor, MI, United States) through a 20x air objective inside the incubator at 37°C in 5% CO_2_. The system is equipped for phase contrast and two fluorescent channels (green excitation 440–480 nm, emission 504–544 nm and red excitation 565–605 nm, emission 625–705 nm), the Basler scout scA1400-30gm monochrome camera and IncuCyte Zoom software to remotely control the imaging and further measure and analyze the fluorescence intensities for captured images. JC-1 dye exhibit MMP-dependent accumulation in mitochondria, indicated by a fluorescence shift between red and green. The color change is due to formation of red fluorescent JC-1-aggregates in energized mitochondria, green monomeric JC-1 dye in depolarized organelles, and orange that indicate polarized mitochondria. The level of mitochondrial polarization were calculated as the ratio between red and green fluorescence intensities, which is dependent on the MMP. Values were normalized to the diluent control (100%) and data represented the mean ± SE. At least six independent experiments were done on separate days on different cell passages.

### Isolation of a Mitochondria-Enriched Protein Fraction

Cells grown in flasks were washed twice with PBS, pH 7.3, harvested and centrifuged at 300 *g* for 7 min at RT. The pellets containing cells were resuspended in lysis buffer (250 mM sucrose, 10 mM Trizma Base, 1 mM EGTA, pH 7.4) on ice and lysed using a Potter-Elvehjem pestle homogenizer by treating the sample with 20 pestle strokes on ice. The lysates were further centrifuged at 600 *g* for 10 min at 4°C. The pellets containing cell debris were discarded, and collected supernatants were centrifuged at 600 *g* for 10 min at 4°C; this step was repeated several times until no pellets were visible. Finally, the supernatants were centrifuged at 7,000 × *g* for 10 min at 4°C and the resulting pellets containing mitochondria were frozen at −20°C.

### In-solution Digestion

The frozen pellets containing mitochondria were thawed and incubated with 50 μl 0.1% (w/v) RapiGest (Waters, Milford, MA, United States) in 50 mM ammonium bicarbonate for 3 min at 90°C. The samples were cooled to RT, reduced in 5 mM DTT for 30 min at 60°C and alkylated with 10 mM IAA for 30 min at RT in dark. MS-grade trypsin (Thermo Scientific) was used for the following enzymatic digestion according to the manufacturer’s recommendations. Pierce C18 tips (Thermo Scientific) were used to desalt the obtained peptides. Peptides were then reconstituted in 0.1% formic acid in milliQ water and peptides concentrations were estimated by A280 was measured (NanoDrop ND-1000 Spectrophotometer, Thermo) prior to liquid chromatography tandem mass spectrometry (LC-MS/MS) analyses.

### LC-MS/MS

Peptides were separated by reverse phase chromatography on a 20 mm × 100 μm C18 pre column followed by a 100 mm × 75 μm C18 column with particle size 5 μm (NanoSeparatoons, Nieuwkoop, Netherlands) at a flow rate of 300nL/min with EASY-nLC II (Thermo Scientific) by a gradient of 0.1% formic acid in water (A) and 0.1% formic acid in acetonitrile (B) as follows: from 2% B to 35% B in 70 min; from 35% B to 100% B in 20 min. Automated online analyses were performed with a LTQ Orbitrap Velos Pro hybrid mass spectrometer (Thermo Scientific) with a nano-electrospray source. The top 20 most intense multiply charged ions were selected with an isolation window of 2.0 and fragmented in the linear ion trap by collision-induced dissociation with a normalized collision energy of 35%. Dynamic exclusion of sequenced peptides for 60s and charge state-filtering disqualifying singly charged peptides were activated and predictive AGC was enabled.

### Database Searching

The generated raw files were analyzed using Sequest HT in Proteome Discoverer (Thermo Scientific, San Jose, CA, United States CS version 1.4.0.288) using complete *Homo sapiens* and *Mus musculus* protein sequence databases available at UniProt^2^. Proteins were identified with the following parameters: trypsin as a digestion enzyme; maximum number of missed cleavages 2; fragment ion mass tolerance 0.60 Da; parent ion mass tolerance 10.0 ppm; carbamidomethylation of cysteine as fixed modification.

### Data Evaluation and Label-Free Quantification

Identified proteins were validated using the SCAFFOLD software (Version 4.4.8; Proteome Software, Inc., Portland, OR, United States). Identifications were based on a minimum of two peptides, minimum 95% peptide identification probability, and minimum 99% protein identification probability (Nesvizhskii et al., 2003). Proteins, that contained similar peptides, and which could not be differentiated based on MS/MS analysis alone, were grouped into clusters to satisfy the principles of parsimony. The label-free quantitative analysis was performed using emPAI and NSAF to calculate the relative protein abundance of each protein cluster in the sample; normalization was performed in order to account for variations between samples (Zybailov et al., 2006). Quantitative differences were statistically analyzed by ANOVA and two-tailed Student’s *t*-test; differences with *P*-values lower than 0.05 were considered statistically significant.

### Bioinformatics Analysis

The interactions between the identified proteins were analyzed by the Search Tool for the Retrieval of Interacting Genes and Proteins, STRING 10.0^3^ using medium confidence score 0.4 and all active interaction sources (Franceschini et al., 2013). STRING and NCBI GO annotations by SCAFFOLD analyses were used to group proteins into functional classes.

### Statistical Analyses

Data in the graphs are presented as mean ± SE, and statistical analyses are based on a paired two-tailed Student’s *t*-test. *P*-values < 0.05 (^∗^), < 0.01 (^∗∗^), and < 0.001 (^∗∗∗^) were considered significant. This is also specified in the figure legends. For mitochondrial energetics, at least three independent experiments were done in 3–5 replicates and on separate days on different cell passages. The experiments on TEM and MMP were done 3 and 6 times, respectively, on separate days and different cell passages. The experiments for proteome were repeated at least six times on separate days, and data evaluation, quantification, and statistical analyses are described above.

## Results

### 3O-C_12_-HSL Induces Changes in Mitochondrial Dynamics and Architecture at Nanoscale Resolution

**FIGURE 2:**
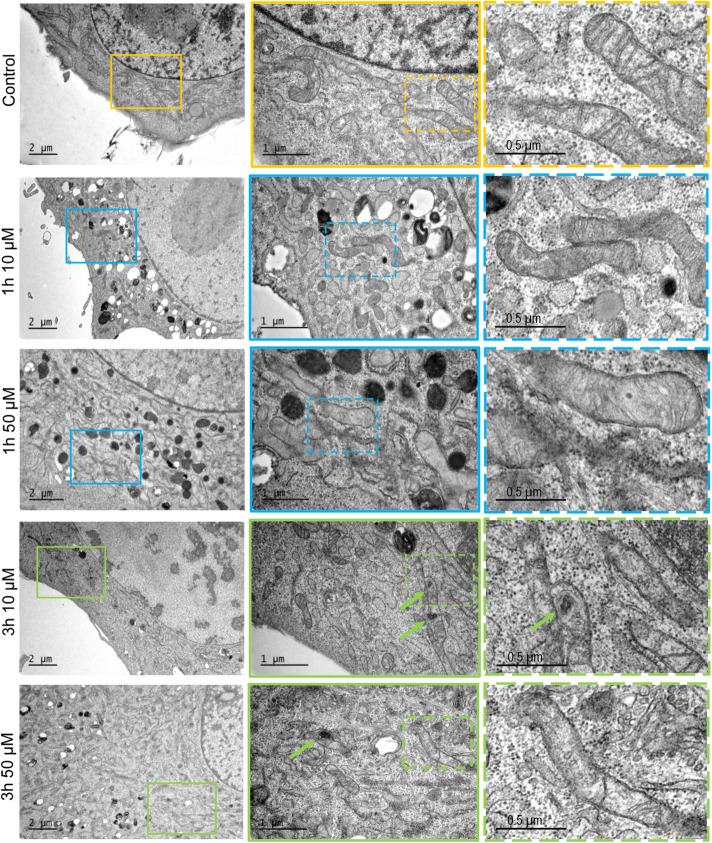
Visualization of mitochondria and their ultrastructure in fibroblasts treated with 3O-C_12_-HSL. Cells were either treated with 0.02% DMSO as a diluent (Control), or 10 and 50 μM 3O-C_12_-HSL for 1 or 3 h. Samples were fixed, stained with uranyl acetate and imaged by TEM. The data are from one of three independent experiments. Left panel, bar: 2 μm. Middle panel, bar: 1 μm. Right panel, bar: 0.5 μm. Size of zoomed inserts in the right panel is 1.3 μm × 1.5 μm.

**FIGURE 3:**
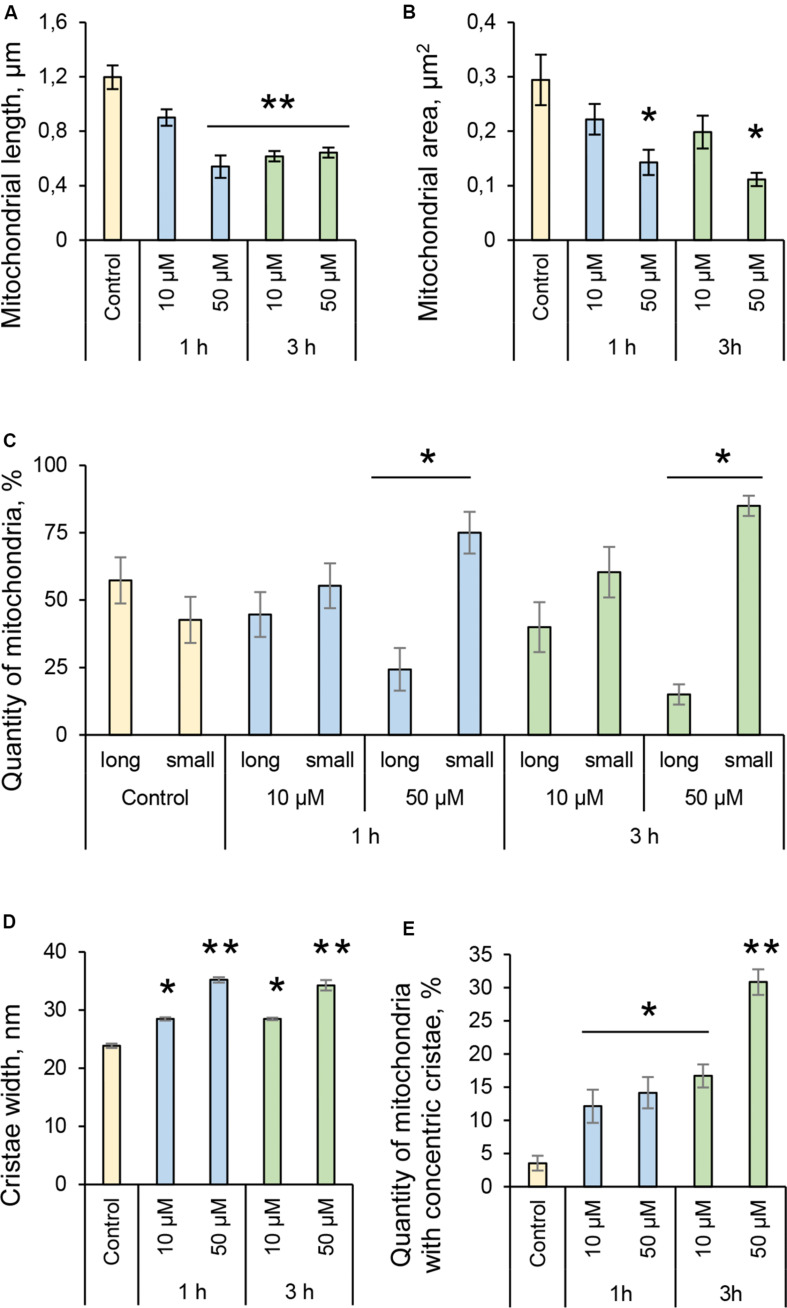
Quantification of mitochondria dynamics and ultrastructure in fibroblasts in response to 3O-C_12_-HSL. **(A)** Mitochondrial length, μm. **(B)** Mitochondrial area, μm^2^. **(C)** Quantity of mitochondria, %. Long organelles were considered as > 0,2 μm^2^ and small as < 0,2 μm^2^. **(D)** Cristae width, nm. **(E)** Quantity of mitochondria with concentric cristae, % of control. Columns represent the mean ± SE. Significant differences are indicated with *P < 0.05 and **P < 0.01, as analyzed by two-tailed Student’s *t*-test. Data from three different experiments performed on separate days on different cell passages, and at least 300 mitochondria in different cells per condition were analyzed.

**FIGURE 4:**
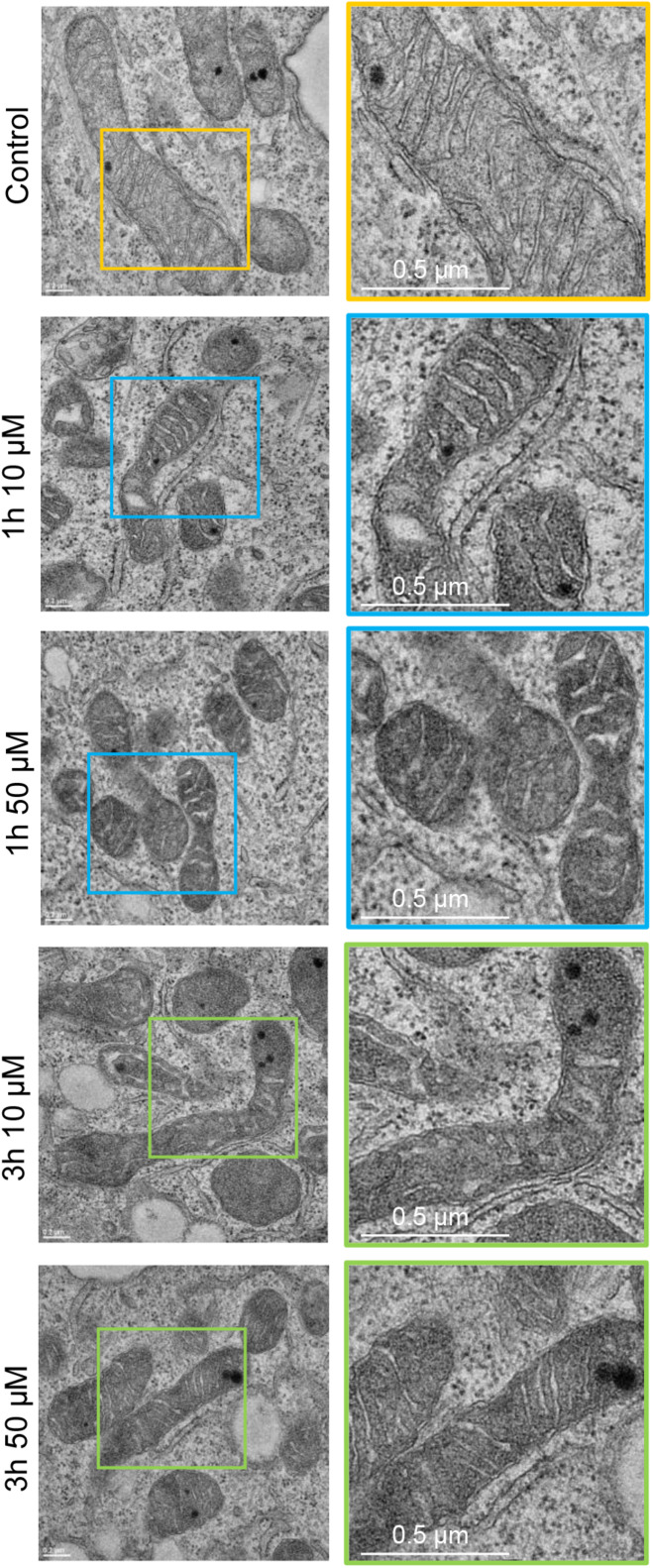
Visualization of mitochondria and their ultrastructure in 3O-C_12_-HSL-treated epithelial Caco-2 cells. Cells were either treated with 0.02% DMSO as a diluent (Control), or 10 and 50 μM 3O-C_12_-HSL for 1 or 3 h. Cells were fixed, stained with uranyl acetate and analyzed by TEM. The data are from one of three independent experiments. Left panel, bar: 0.2 μm. Right panel, bar: 0.5 μm. Size of zoomed inserts with colored boards in the right panel is approximately 1 μm × 1 μm.

**FIGURE 5:**
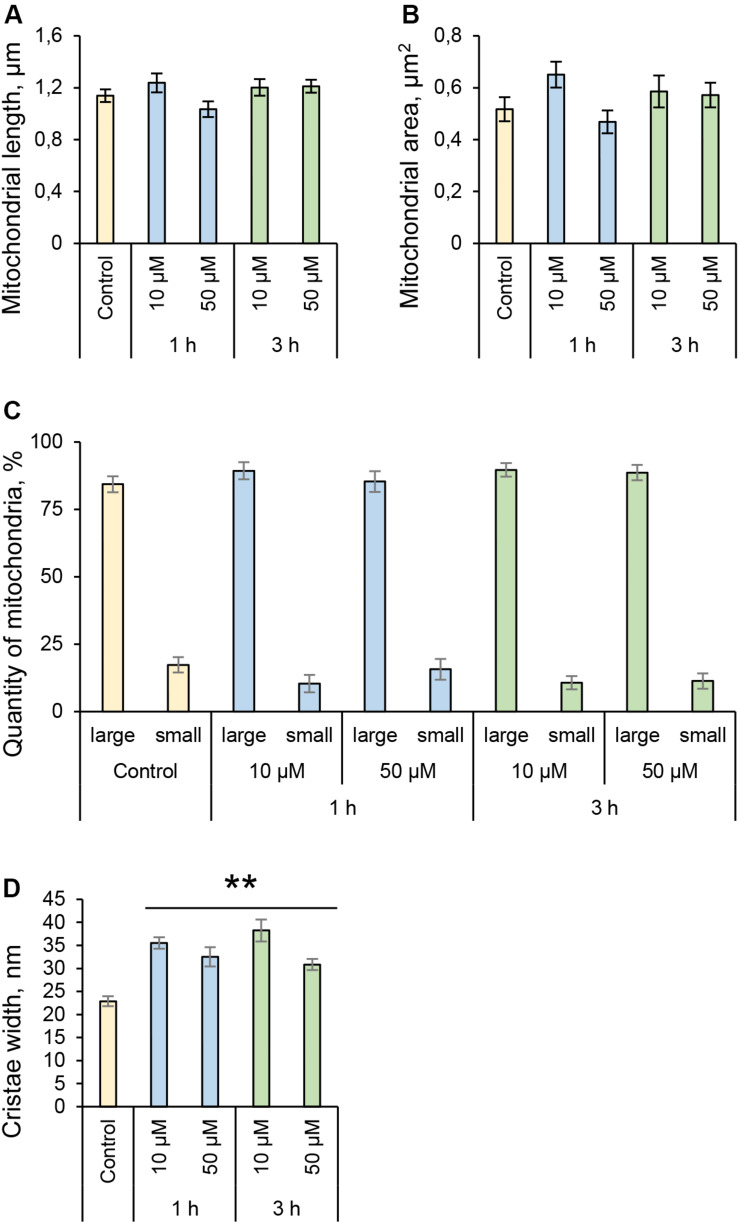
Quantification of mitochondria dynamics and ultrastructure in epithelial Caco-2 cells stimulated with 3O-C_12_-HSL. **(A)** Mitochondrial length, μm. **(B)** Mitochondrial area, μm^2^. **(C)** Quantity of mitochondria, %. Large organelles were considered as >0,2 μm^2^ and small as <0,2 μm^2^. **(D)** Cristae width, nm. Columns represent the mean ± SE. Significant differences are indicated with ** when *P* < 0.01, as analyzed by two-tailed Student’s *t*-test. Data from three different experiments performed on separate days on different cell passages, and at least 300 mitochondria in different cells per condition were analyzed.

Many pathogenic microorganisms modulate host mitochondrial structure and function in order to maintain their own replicative niche or to favor dissemination (Rudel et al., 2010; Ashida et al., 2011). Therefore, we employed nanoscale resolution imaging by TEM to investigate whether 3O-C_12_-HSL affected different ultrastructural characteristics of mitochondria in fibroblasts (Figure 2) and epithelial cells (Figure 4). We thus quantified observed changes including the mitochondrial length and area, proportion of elongated and small mitochondria, and cristae width (Figures 3, 5). In the control groups of either cell type mitochondria were mostly elongated (Figures 3A–C, 5A–C) and showed structured, regular and deeply folded cristae (Figures 3D, 5D). We also observed that treatment of fibroblasts with 50 μM 3O-C_12_-HSL for 1 or 3 h (in contrast to 10 μM and the control) resulted in a rapid fragmentation of mitochondrial network, as evidenced by decreased mitochondrial length and area (Figures 3A,B) and an elevated number of small mitochondria (Figure 3C). When treated with 10 or 50 μM 3O-C_12_-HSL, as compared with control, fibroblasts displayed irregular mitochondrial cristae with shorter folding throughout the matrix, significantly increased cristae width (Figure 3D) and number of organelles with multi-folded, concentric cristae (Figure 3E). By contrast, treatment of epithelial cells with 3O-C_12_-HSL did not affect mitochondrial size (Figures 5A,B) or the balance between large and small organelles (Figure 5C); these structural patterns of mitochondrial network were near to control. In terms of the mitochondrial membrane ultrastructure, 3O-C_12_-HSL-treated epithelial cells still maintained deeply folded cristae throughout matrix, however their width was increased (Figure 5D). It should be noted that cell viability of both fibroblasts and epithelial cells treated with 3–100 μM 3O-C_12_-HSL for 4–72 h remained similar to control (Supplementary Figure S1 and Method S1). Together, these high-resolution imaging data demonstrate that 3O-C_12_-HSL signaling molecules can provoke changes in mitochondrial dynamics and disrupts their inner membrane ultrastructure and that there are distinct cell-type specific details and strength of these effects. In particular, intestinal epithelial cells were less affected compared with fibroblasts (Figure 9).

### 3O-C_12_-HSL Disrupts Mitochondrial Respiration and Energetics

**FIGURE 6:**
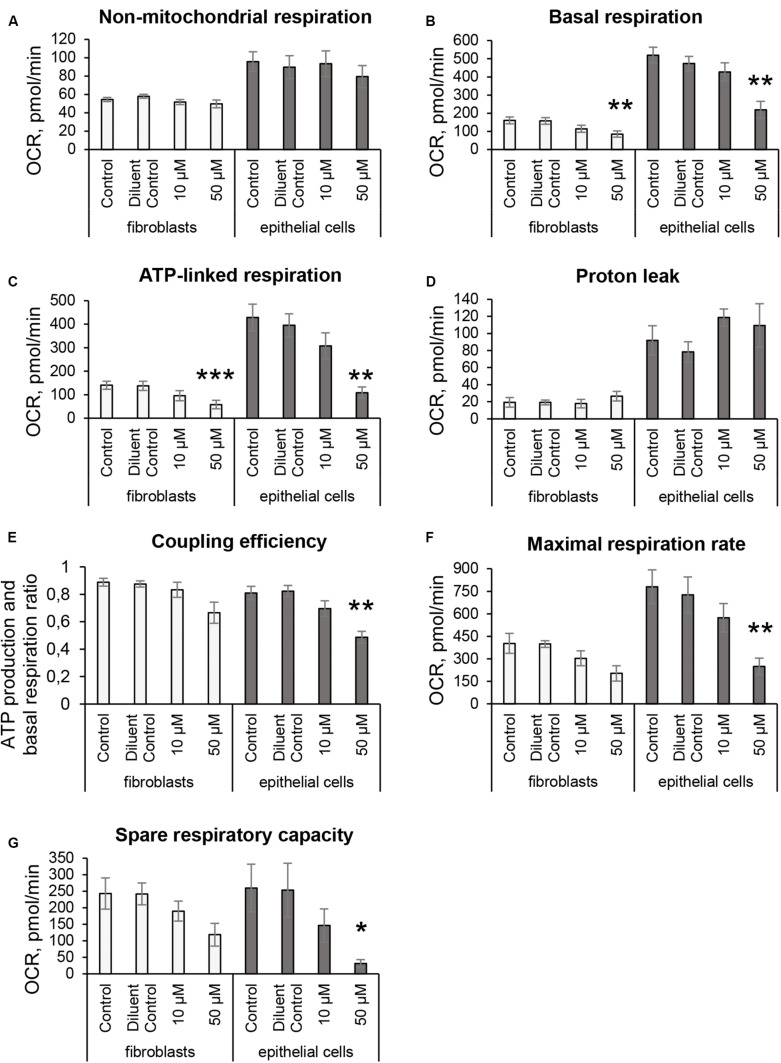
Mitochondrial respiratory activity in fibroblasts and intestinal epithelial Caco-2 cells in response to 3O-C_12_-HSL. Cells were either untreated (Control) or treated with 0.02% DMSO as a diluent (Diluent Control), or 10 and 50 μM 3O-C_12_-HSL for 3 h and then analyzed using the Seahorse XF24 Analyzer and Cell Mito Stress Test assay to study key characteristics of respiration and mitochondrial function. OCR levels (pmol/min) were measured in real time, first as baseline, and then in response to adding modulators (as shown in Figures 1A,B) that target different components of ETC in the mitochondria. **(A)** Non-mitochondrial respiration. **(B)** Basal respiration. **(C)** ATP-linked respiration. **(D)** Proton leak. **(E)** Coupling efficiency. **(F)** Maximal respiration. **(G)** Spare respiratory capacity. Columns represent the mean ± SE. Significant differences are indicated with *P < 0.05, **P < 0.01, and ***P < 0.001, as analyzed by a paired two-tailed Student’s *t*-test, compared to the diluent control. White columns represent data on fibroblasts based on three independent experiments done in 3–5 replicates and on separate days with different cell passages. Gray columns represent data on intestinal epithelial monolayers based on five independent experiments done in 3–5 replicates on separate days with different cell passages.

Since 3O-C_12_-HSL-treatment caused remodeling of mitochondrial size, morphology and membrane ultrastructure (Figures 2–5), parallel alterations in cellular bioenergetics driven by mitochondria might occur (Brand and Nicholls, 2011; Kuhlbrandt, 2015). To study this, we utilized the Seahorse XF24 Cell Mito Stress assay, which in real time allows to monitor OCR of living cells in response to modulators that target different components of ETC in mitochondria (Figures 1A,B) and hereby to measure major characteristics of mitochondrial function in fibroblasts and epithelial cells (Figure 6). We detected that the non-mitochondrial OCRs in all groups including control were relatively low, but constant (Figure 6A). This likely reflects the activity of non-mitochondrial enzymes in cellular oxygen uptake and typically, yields approximately 10% of the total rates in mammalian cells (Nobes et al., 1990). After treatment with 50 μM 3O-C_12_-HSL for 3 h, but not with 10 μM, we observed a significant decrease in basal mitochondrial respiration (Figure 6B) and ATP-linked respiration (Figure 6C) in both fibroblasts and epithelial cells, as compared to the diluent control. In epithelial monolayers, coupling efficiency, maximal respiration and spare respiratory capacity were also significantly decreased while these parameters only tended to be slightly lower in fibroblasts (Figures 6E–G). However, 3O-C_12_-HSL did not affect proton leak across the mitochondrial membrane in the presence of oligomycin, as compared to the diluent control (Figure 6D). Typically, the basal respiration is strongly regulated by ATP turnover and partly by proton leak and substrate oxidation (Brand and Nicholls, 2011). Here, changes caused by 3O-C_12_-HSL were mainly controlled by ATP-linked oligomycin-sensitive respiration and less by oligomycin-insensitive respiration that reflect proton leak. Coupling efficiency is the fraction of basal mitochondrial respiration used for ATP synthesis and may vary with ATP demand; this serves as an additional sensitive and internally normalized index reflecting mitochondrial disfunction in 3O-C_12_-HSL-treated epithelial cells (Figure 6E). The maximum respiration rate caused by addition of the protonophore uncoupler FCCP reflects stimulated mitochondrial respiration. Spare respiratory capacity is assessed by the difference between maximum respiration rate and basal respiration. A decrease in maximum respiration rate and spare respiratory capacity are yet other indicators of mitochondrial function compromised by 3O-C_12_-HSL in epithelial monolayers (Figures 6F,G). Taken together, these findings show that 3O-C_12_-HSL impairs mitochondrial respiration in dose-dependent manner in both fibroblasts and epithelial cells. Notably, 3O-C_12_-HSL achieved a more prominent effect on the bioenergetics of epithelial cells compared with fibroblasts, targeting five versus two of the seven major parameters of mitochondria respiration (Figure 9).

### 3O-C_12_-HSL Decreases Mitochondrial Membrane Potential

**FIGURE 7:**
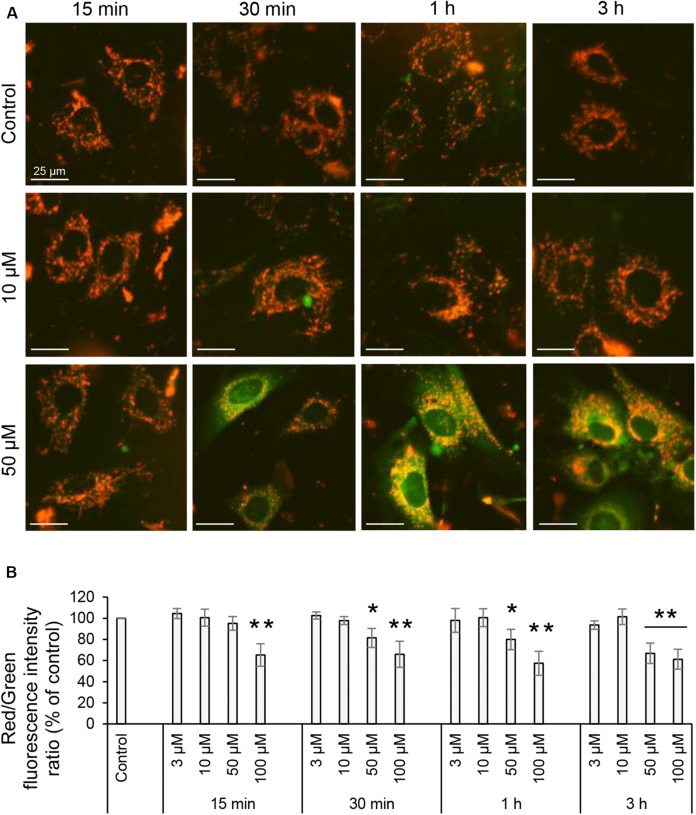
Mitochondrial membrane potential of living fibroblasts induced with 3O-C_12_-HSL. **(A)** Cells were either treated with 0.02% DMSO as a diluent (Control), or exposed to 3, 10, 50, or 100 μM 3O-C_12_-HSL and then stained with sensor JC-1 that exhibit MMP-dependent accumulation in mitochondria, indicated by a fluorescence shift between red and green. Living cells were visualized using IncuCyte Zoom live cell imaging and analysis system to measure fluorescence intensities. All images are merged between red (energized mitochondria) and green (depolarized mitochondria) fluorescence; orange indicate polarized mitochondria. Image size is 100 μm × 100 μm. Bar: 25 μm. **(B)** Quantification of the mitochondrial polarization. The level of mitochondrial polarization was calculated as the ratio between red and green fluorescence intensities, and values were normalized to the diluent control (100%). Columns represent the mean ± SE for the ratio between red and green fluorescence intensities. Significant differences are indicated with *P < 0.05 and **P < 0.01, as analyzed by two-tailed Student’s *t*-test. Data from at least six independent experiments performed on separate days on different cell passages, and at least 1500 cells per condition were analyzed.

**FIGURE 8:**
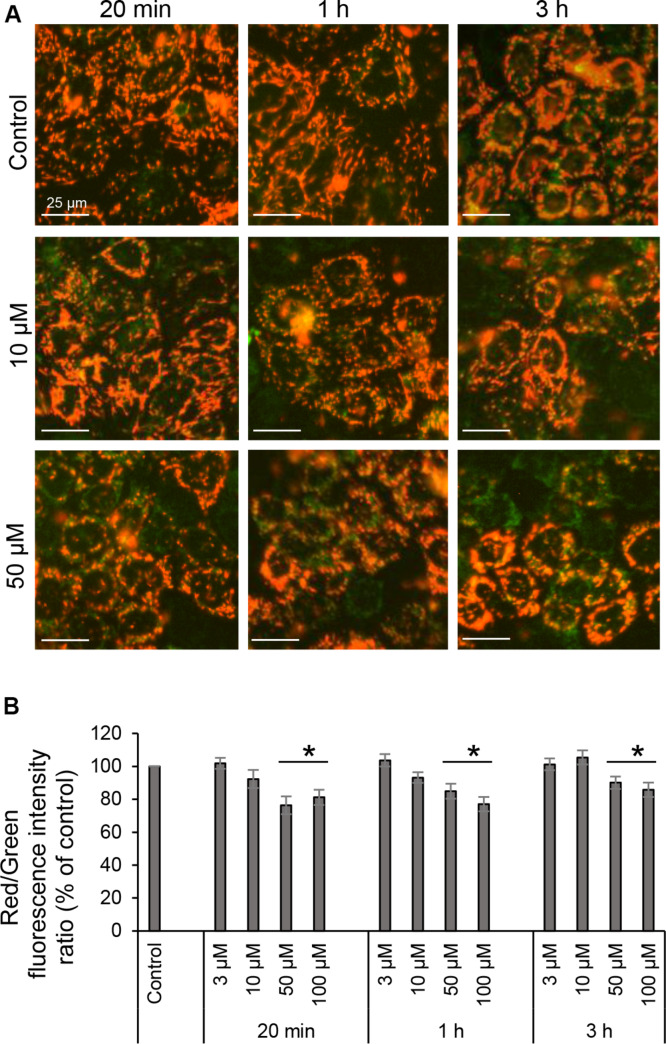
Mitochondrial membrane potential of living intestinal epithelial cells in response to 3O-C_12_-HSL. **(A)** Cells were either treated with 0.02% DMSO as a diluent (Control), or exposed to 3, 10, 50, or 100 μM 3O-C_12_-HSL and then stained with sensor JC-1 that exhibit MMP-dependent accumulation in mitochondria, indicated by a fluorescence shift between red and green. Living cells were visualized using IncuCyte Zoom live cell imaging and analysis system to measure fluorescence intensities. All images are merged between red (energized mitochondria) and green (depolarized mitochondria) fluorescence; orange indicate polarized mitochondria. Image size is 100 μm × 100 μm. Bar: 25 μm. **(B)** Quantification of the mitochondrial polarization. The level of mitochondrial polarization was calculated as the ratio between red and green fluorescence intensities, and values were normalized to the diluent control (100%). Columns represent the mean ± SE for the ratio between red and green fluorescence intensities. Significant differences are indicated with *P < 0.05, as analyzed by two-tailed Student’s *t*-test. Data from at least six independent experiments performed on separate days on different cell passages, and at least 1000 cells per condition were analyzed.

**FIGURE 9:**
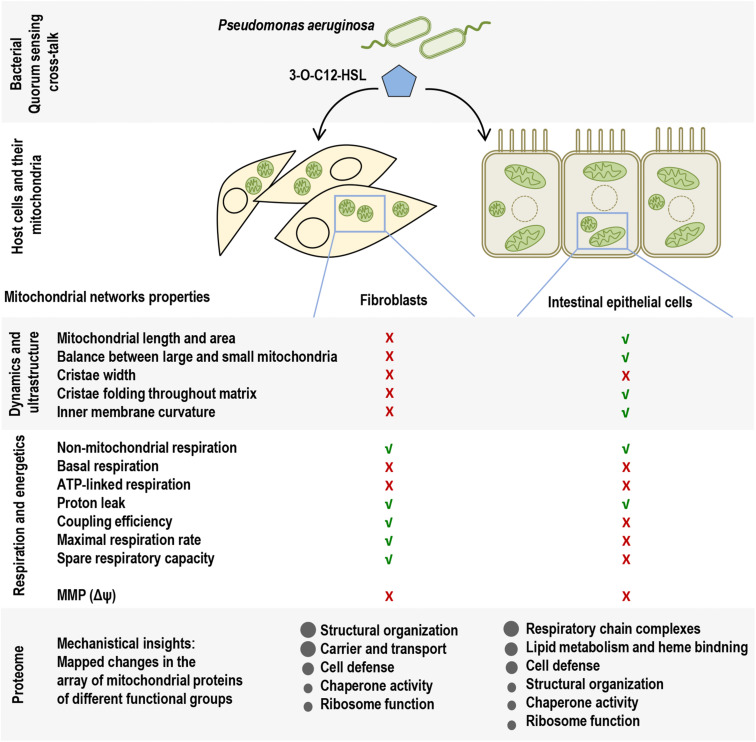
Overview of the study on the impact of *P. aeruginosa* QS molecule 3O-C_12_-HSL on mitochondrial morphology, energetics, and proteome in host cells. In the upper panel, the scheme illustrates main QS molecule 3O-C_12_-HSL (blue pentagon) of *P. aeruginosa* that together with other signals in the QS system provides cell–cell communication between bacteria and control biofilm development, virulence traits, adaptation and interactions with the host. QS molecules, like 3O-C_12_-HSL, can modulate the behavior of host cells and their mitochondrial networks, e.g., in the two types of cells used in this study, fibroblasts and intestinal epithelial cells. In the middle panels, the scheme summarizes multifactorial impact of 3O-C_12_-HSL on mitochondrial dynamics, ultrastructure, respiration and energetics, and that there are distinct cell-type specific details and strength of these effects. Mitochondrial properties and functions similar to those in the controls are depicted by green check mark, and those that were affected or disrupted depicted with red cross. The lower panel reflects mechanistical insights and mapped changes in a common and cell-type specific array of components in the mitochondrial proteome.

Proton pumps of the ETC in the mitochondria, mainly complexes I, III, and IV generate the MMP (Figure 1B), providing a driving force for mitochondrial ATP synthesis and transport of charged compounds essential for mitochondrial homeostasis (Brand and Nicholls, 2011). Since respiration and energetics driven by mitochondria were decreased in response to 3O-C_12_-HSL, we decided to further investigate whether the level of MMP were affected. This was analyzed by imaging of MMP using JC-1 sensor in living fibroblast (Figure 7A) and epithelial Caco-2 cells (Figure 8A) with further quantification of ratio between red and green fluorescence intensity (Figures 7B, 8B). We detected a significant decrease in MMP in fibroblasts treated with 3O-C_12_-HSL in a dose- and time-dependent manner, in clear contrast to control cells (Figures 7A,B). The drop of MMP induced by 3O-C_12_-HSL in the epithelial monolayers also displayed a distinct time- and dose-dependent pattern, which however was not so dramatic as in the fibroblasts (Figures 8A,B). Thus, 3O-C_12_-HSL-treatment resulted in depletion of MMP in both cell types supporting perturbed mitochondrial function (Figure 9).

### Alterations in the Mitochondrial Proteome in Response to 3O-C_12_-HSL

It is well-established that proteins residing in mitochondria are important components of the mechanisms involved in regulation of mitochondrial homeostasis and numerous essential functions (Kuhlbrandt, 2015). We thus further aimed to identify differentially expressed mitochondrial proteins of fibroblasts and epithelial cells in response to *P. aeruginosa* 3O-C_12_-HSL. We performed LC-MS/MS based quantitative proteomic analyses of the mitochondria-enriched fractions obtained from fibroblasts and epithelial monolayers, which were subjected to three different conditions, i.e., exposed to 10 or 50 μM 3O-C_12_-HSL for 4h or treated with DMSO as a diluent control. Out of more than 1000 protein clusters identified in each sample, around 300 proteins clusters were annotated as mitochondrial according to NCBI GO annotations, which represents a good enrichment degree in our samples with about 25–30% coverage of the annotated mitoproteome, as the total mammalian proteome contains only about 7–8% mitochondrial proteins. The rest of proteins could be mainly associated with other organelles (ER and nucleus), the cytoskeleton and the cytoplasm; the categories of subcellular components were obtained from NCBI annotations, where each protein can have multiple subcellular locations. The molecular weight of the proteins varied between 6 and 693 kDa (not shown). Using ANOVA and two-tailed Student’s *t*-test, we identified four mitochondrial proteins appearing at similar expression levels in each sample, which represents a good enrichment quality and reflects an equal amount of mitochondria: voltage-dependent anion-selective channel protein 1, mitochondrial import receptor subunit TOM20, cytochrome c oxidase subunit 4 and 60 kDa heat shock protein (not shown).

Further, we identified proteins which were differentially expressed after treatment with 10 or 50 μM 3O-C_12_-HSL for 4 h, in comparison to control, using ANOVA (Supplementary Tables S1, S2) and two-tailed Student’s *t*-test (Supplementary Tables S3–S6), and differences with *P*-values lower than 0.05 were considered statistically significant. To find proteins that could impact mitochondrial dynamics and homeostasis, we further focused on the genuine mitochondrial proteome according to Search Tool for the Retrieval of Interacting Genes and Proteins (STRING) and NCBI GO annotations. Thus, differentially expressed mitochondrial proteins in 3O-C_12_-HSL-treated fibroblasts and epithelial cells were identified (Tables 1, 2) and included in the further bioinformatics analyses. Based on STRING and NCBI GO analyses in fibroblasts, the differentially expressed mitochondrial proteins could be allocated in the functional groups involved in carrier and transport, structural organization, cell defense and response to stress (Table 1). Most notably, the inner membrane ADP/ATP translocase 2, DnaJ, metaxin-2, prohibitin, and thioredoxin-dependent peroxide reductase were significantly altered after 3O-C_12_-HSL-treatment in fibroblasts (Table 1). In epithelial cells, differentially expressed mitochondrial proteins could be organized into groups responsible for respiratory chain complexes, cell defense and chaperone activity, lipid metabolism and heme binding, and structural organization (Table 2). Three proteins of the mitochondrial ETC complexes: succinate CoA ligase, succinate dehydrogenase assembly factor 2 and cytochrome c oxidase were downregulated upon 3O-C_12_-HSL-treatment (Table 2). This is an additional issue in our data on 3O-C_12_-HSL-induced disruption of mitochondrial energetics (Figure 6). In parallel, three proteins of ETC complexes, such as NADH dehydrogenase, NAD transhydrogenase, and cytochrome c1 heme protein become more abundant in response to 50 μM 3O-C_12_-HSL (Table 2 and Supplementary Table S6). Thus, key ETC complexes were dysregulated after 3O-C_12_-HSL-treatment of epithelial Caco-2 monolayers. Our data point to an altered expression of two proteins valuable for the MICOS, isoform 2 of MICOS complex subunit MIC60 and periplakin (Table 2). This corroborates our findings on ultrastructure, where we showed that 3O-C_12_-HSL-treated epithelial cells still maintained deeply folded cristae throughout matrix, albeit their width was significantly increased (Figure 5D). We also observed that 3O-C_12_-HSL perturbed the expression of mitochondrial proteins associated with lipid metabolism and heme binding: prostaglandin E synthase 2 and cytochrome c1 heme protein (Table 2). Indeed, fatty acid metabolism and heme binding contribute to establishment of a bioenergetically favorable environment during infection (Rastogi et al., 2019). A group of proteins became differentially abundant in response to 3O-C_12_-HSL were those with chaperone activity and involved in cell defense and response to stress: stress-70 protein, GrpE protein homolog 1, peroxiredoxin-5 (Table 2), DnaJ homolog subfamily A member 1 and thioredoxin-dependent peroxide reductase (Table 1). Finally, an altered expression status of proteins associated with mitochondrial translation both in fibroblasts (39S ribosomal protein L38) and epithelial cells (isoform 2 of 39S ribosomal protein L47) was also noticed (Tables 1, 2). In summary, 3O-C_12_-HSL has a profound and cell-type specific effect on mitochondrial proteins that belong to different functional groups and this is in line with our data on mitochondrial architecture and bioenergetics (Figure 9).

**TABLE 1:** Differentially expressed mitochondrial proteins in fibroblasts after treatment with 10 or 50 μM 3O-C_12_-HSL for 4 h compared to the diluent control.

**Identified proteins**	**UniProt accession**	**MW kDa**	***P*-value emPAI ANOVA**	**Quantitative profile**			**Functional groups #**				
				**Control**	**10 μM**	**50 μM**					
DnaJ homolog subfamily A member 1	DNJA1_MOUSE	45	0.012	High	High	Low	**X**		**X**		**X**
Cluster of ADP/ATP translocase 2	ADT2_MOUSE	33	0.011	High	Low	High	**X**				
Prohibitin	PHB_MOUSE	30	0.0043	Low	High	Low		**X**			
Metaxin-2	MTX2_MOUSE	30	0.024	Low	High	Low	**X**	**X**			
39S ribosomal protein L38, mitochondrial	RM38_MOUSE	45	0.05	Low	High	Low				**X**	
Thioredoxin-dependent peroxide reductase, mitochondrial	PRDX3_MOUSE	28	0.042	High	Low	Low		**X**	**X**		

*# Functional groups of human proteins: **X** – carrier and transport; **X** – structural organization; **X** – cell defense and response to stress and pathogens; **X** – ribosomal function; **X** – chaperone activity. STRING and SCAFFOLD analyses were used to group proteins into functional classes. Data are from six independent experiments.*

**TABLE 2:** Differentially expressed mitochondrial proteins in Caco-2 epithelial monolayers after treatment with 10 or 50 μM 3O-C_12_-HSL for 4 h compared to the diluent control.

**Identified proteins**	**UniProt accession**	**MW kDa**	***P*-value emPAI ANOVA**	**Quantitative profile**			**Functional groups #**					
				**Control**	**10 μM**	**50 μM**						
Peroxiredoxin-5, mitochondrial	PRDX5_HUMAN	22	0.0024	Low	High	High		**X**				
NADH dehydrogenase [ubiquinone] 1 alpha subcomplex subunit 8	NDUA8_HUMAN	20	0.011	Low	High	High	**X**					
Periplakin	K7EKI8_HUMAN	204	0.021	Low	High	High			**X**			
Prostaglandin E synthase 2	PTGES2_HUMAN	42	0.039	Low	High	High				**X**		
Isoform 2 of 39S ribosomal protein L47, mitochondrial	RM47_HUMAN	27	0.041	High	High	Low						**X**
Peroxisomal bifunctional enzyme	ECHP_HUMAN	79	0.02	High	Low	High				**X**		
Cytochrome c1, heme protein, mitochondrial	CY1_HUMAN	35	0.04	High	Low	High	**X**			**X**		
Cluster of NAD transhydrogenase	NNTM_HUMAN	114	0.024	Low	Low	High	**X**					
GrpE protein homolog 1, mitochondrial	GRPE1_HUMAN	24	0.034	Low	Low	High		**X**			**X**	
Cluster of isoform 2 of MICOS complex subunit MIC60	MIC60_HUMAN	83	0.035	Low	Low	High			**X**			
Stress-70 protein, mitochondrial	GRP75_HUMAN	74	0.041	Low	Low	High		**X**			**X**	
Succinate–CoA ligase [ADP/GDP-forming] subunit alpha, mitochondrial	SUCA_HUMAN	36	0.0078	High	Low	Low	**X**					
Succinate dehydrogenase assembly factor 2, mitochondrial	F5GYJ5_HUMAN	18	0.0091	High	Low	Low	**X**					
Cytochrome c oxidase subunit 5B, mitochondrial	COX5B_HUMAN	14	0.012	High	Low	Low	**X**					

*# Functional groups of human proteins: **X** – respiratory chain complexes; **X** – cell defense and response to stress and pathogens; **X** – structural organization; **X** – lipid metabolism and heme binding; **X** – chaperone activity; **X** – ribosomal function. STRING and SCAFFOLD analyses were used to group proteins into functional classes. Data are from six independent experiments.*

## Discussion

Intracellular and extracellular bacterial pathogens, including *P. aeruginosa*, interfere and manipulate with mitochondrial homeostasis to create an environment comfortable for their survival, replication, and spreading. Thus, pathogens can modulate mitochondrial function to favor their own spreading by inducing host cell apoptosis. Alternatively, bacteria can maintain their replicative intracellular niche by preventing apoptosis, shutting down mitochondrial energetics or interfering with mitochondrial innate immune signaling (Rudel et al., 2010; Ashida et al., 2011; Stavru et al., 2011; Jabir et al., 2015; Kang et al., 2018).

In this study, we focused on whether and how *P. aeruginosa* 3O-C_12_-HSL affect mitochondria in two distinct types of host cells, using as models the mouse embryonal fibroblasts and human intestinal epithelial Caco-2 cells. The choice of cell models is assumed to represent the different type of cells rather than their species, since both are mammalian, where their respective genes and proteins generally share the same functions. Mouse-specific gene clusters include genes involved in reproduction, olfaction, gustation, and host defense and immunity, like certain genes for MHC and G-protein-coupled receptors (Emes et al., 2003).

When applied to mammalian cells, hydrophobic AHL can quickly diffuse across the plasma membrane via interaction with phospholipids (Davis et al., 2010) to access the cytoplasm, ER and mitochondria (Guo et al., 2020). The absorption of AHL into the host cells may further be enhanced by cell membrane extensions and protrusions, such as microvilli on the apical surface of differentiated polarized intestinal epithelial cells. Thus, bearing dense and tightly packet microvilli, gut epithelial cells enlarge their surface up to 100 times, and hereby also increase the potential exchange of substances between external environment and epithelial monolayers. Fibroblasts being activated to migration became polarized in the direction of motion possessing leading and trailing edges, and thin membrane protrusions, lamellipodium and filopodia that can increase cell substratum adhesion. The entry of 3O-C_12_-HSL is also facilitated by membrane microdomains (Davis et al., 2010) that interact with a sub-membrane organization of filamentous actin, actin-binding proteins and anchoring membrane proteins. The uptake is followed by recognition of 3O-C_12_-HSL by the IQ-motif-containing GTPase-activation protein IQGAP1 (Karlsson et al., 2012b) and nuclear peroxisome proliferator-activated receptors (Jahoor et al., 2008). 3O-C_12_-HSL does not interact with membrane-bound Toll-like receptors expressed on immune cells (Kravchenko et al., 2006).

Here, using the model of fibroblasts and intestinal epithelial cells and employing high-resolution imaging we show that *P. aeruginosa* 3O-C_12_-HSL signaling molecules cause changes in mitochondrial dynamics and their membrane ultrastructure and that these effects have distinct cell-type specific details (Figure 9). Thus, in 3O-C_12_-HSL-treated fibroblasts we observed a rapid fragmentation of mitochondrial network, as evidenced by a decreased mitochondrial size and an elevated number of small organelles. A reduction of mitochondrial size has also previously been observed in bronchial epithelial cells (Maurice et al., 2019). Moreover, we demonstrated that in response to 3O-C_12_-HSL, mitochondria in fibroblasts displayed irregular cristae with shorter folding throughout matrix, increased cristae width and enhanced quantity of organelles with multi-folded, concentric cristae. In contrast, in intestinal epithelial monolayers, 3O-C_12_-HSL did not affect mitochondrial size and dynamics; cristae were still deeply folded throughout matrix, albeit more open and wider. Thus, our present study on mitochondrial morphology demonstrate that 3O-C_12_-HSL causes more prominent changes in mitochondrial dynamics and disruption of their ultrastructure in fibroblasts compared with intestinal epithelial cells (Figure 9).

Alterations in mitochondrial dynamics and ultrastructure likely cause changes in cellular bioenergetics (Kuhlbrandt, 2015). We therefore further focused our investigations on the mitochondrial respiration and metabolic potential. Indeed, we observed that mitochondrial respiration was decreased in response to 3O-C_12_-HSL and paralleled by a drop in MMP. In particular, the bioenergetics of intestinal epithelial cells was more compromised than in fibroblasts (Figure 9). Thus, these data point out that there is a very close relationship between the functional state and ultrastructural organization of the mitochondria in both fibroblasts and intestinal epithelial monolayers, being in line with several earlier observations with other cell types (Schwarzer et al., 2012, 2014; Maurice et al., 2019).

Additionally, 3O-C_12_-HSL has been demonstrated to influence mitochondrial physiology in fibroblasts by releasing cytochrome c via activating caspases 3/7 and 8 without the involvement of Bak/Bax regulators of apoptosis, i.e., having a pro-apoptotic effect (Schwarzer et al., 2014). 3O-C_12_-HSL may trigger apoptosis and cytotoxicity in many type of cells (Tateda et al., 2003; Schwarzer et al., 2012; Zhao et al., 2016; Neely et al., 2018) and this was recently reviewed (Guo et al., 2020). Still, fibroblasts and polarized human epithelial cells used in this study (Supplementary Figure S1) and earlier (Karlsson et al., 2012b; Losa et al., 2015) and human primary neutrophils and macrophages (Karlsson et al., 2012a; Holm et al., 2016), did not display any apoptotic changes or cell death in response to 3O-C_12_-HSL in the used conditions. Still, cell viability results obtained with different assays, cell types and conditions may partly differ. The AlamarBlue assay used in this study is a simple and non-toxic way of cell viability measuring based on resazurin reduction, but it is not known where in the cell this reduction occurs or if it is affected by cell metabolism and mitochondrial activity. 3O-C_12_-HSL triggers calcium signaling (Karlsson et al., 2012a) and leads to alterations in the redox potential that becomes more reduced in ER and more oxidized in the cytosol (Schwarzer et al., 2012), which might also affect the measured cell viability. Each type of cell or tissue has a different metabolic profile and energetic request relying on various metabolic circuits due to their inherently different cell functions and on physiological or pathological conditions. While *in vivo*, many cells require oxygen for respiration, *in vitro* cultured cells often alter their metabolic request and activity switching to glycolysis. Such a metabolic shift can lead to increased susceptibility to mitochondrial toxicants and apoptosis (Andersen and Kornbluth, 2013). Incidentally, the maturity and differentiation state of epithelial cell growing in monolayers might have protected them against pro-apoptotic effect of 3O-C_12_-HSL (Losa et al., 2015), but not prevented loss of barrier integrity and repair potential (Karlsson et al., 2012b) and mitochondrial activities, resulting in compromised cell migration capacity, barrier, and defense function (West et al., 2011; Zhang et al., 2015; Denisenko et al., 2019). Moreover, it is still debated whether the 3O-C_12_-HSL concentrations produced by planktonic bacteria or those growing as biofilms *in vitro* or *in vivo* in tissues are high enough to provoke apoptosis that requires more than 100 μM. Thus, in the respiratory secretions from bacteria-colonized cystic fibrosis patients only nM concentrations of AHL have been detected (Singh et al., 2000; Erickson et al., 2002; Chambers et al., 2005), in contrast to 300–600 μM in biofilms established *in vitro* (Charlton et al., 2000).

To elucidate details on how *P. aeruginosa* 3O-C_12_-HSL affects mitochondria, we mapped changes in the mitochondrial proteome. This is a novel and not previously reported approach. Thus, we employed quantitative LC-MS/MS-based proteomic and bioinformatic analyses of the mitochondria-enriched fractions obtained from fibroblasts and epithelial monolayers. We demonstrated that 3O-C_12_-HSL has profound and cell-type specific effect on mitochondrial proteins belonging to different functional groups, which is in line with our data on mitochondrial architecture and bioenergetics. We also found that a group of mitochondrial proteins, all involved in structural organization, transport and response to stress, were significantly altered in response to 3O-C_12_-HSL in fibroblasts. Among these proteins were ADP/ATP translocase 2, DnaJ, metaxin-2, prohibitin, and thioredoxin-dependent peroxide reductase (Table 1). Mitochondrial ATP is transported to the cytoplasm via specialized transport protein localized in the inner membrane, the ADP/ATP carrier, to provide energy to the cell. Any loss or deficiency of this protein leads to dysfunction in cell metabolism and potentially to various diseases (Clemencon et al., 2013) speaking for reduced mitochondrial energetics in response to 3O-C_12_-HSL (Figures 6, 9). Metaxins are localized in the mitochondrial outer membrane and responsible for protein import and sorting pathways in mammalian mitochondria. Many bacterial virulence traits utilize this route to enter mitochondria and target its functions, i.e., via the enteropathogenic *E. coli* Map, *Neisseria* PorB, and *Acinetobacter baumannii* Omp38 proteins (Kozjak-Pavlovic et al., 2008). Prohibitin complexes play a critical role for mitochondria homeostasis and function (Signorile et al., 2019); they stabilize mitochondrial DNA and protect against stresses and apoptosis (Anderson et al., 2019). Prohibitins are also required for the formation of cristae by stabilizing dynamin-like GTPase, an essential component of the mitochondrial fusion machinery (Merkwirth and Langer, 2009); this could explain the fragmentation of the mitochondrial network that occurred in fibroblasts upon 3O-C_12_-HSL-treatment (Figures 2, 3, 9). Knock-down of the components of MICOS resulted in reorganization of cristae structure observed as concentric onion rings of inner membrane (von der Malsburg et al., 2011), which is also in line with our ultrastructural data on fibroblasts (Figures 2, 3, 9).

Mammalian mitochondria perform the oxygen-dependent oxidation of simple sugars to create ATP, the main energy source in the cells. Five large protein complexes I–V embedded in inner mitochondrial membrane are involved in this process and build together the ETC (Kuhlbrandt, 2015). We found that in 50 μM 3O-C_12_-HSL-treated epithelial cell (Table 2 and Supplementary Table S6), several proteins of complexes II and IV were downregulated (succinate CoA ligase, succinate dehydrogenase assembly factor 2, and cytochrome c oxidase) and that proteins of complexes I and III were upregulated (NADH dehydrogenase, cluster of NAD transhydrogenase and cytochrome c1 heme protein). ATP synthase of complex V was upregulated in fibroblasts in response to 3O-C_12_-HSL (Supplementary Table S4). It has been reported also that 3O-C_12_-HSL can enhance the activity of complexes IV and V in intestinal goblet cell (Tao et al., 2018), suggesting that ETC complexes may undergo different fates in 3O-C_12_-HSL-treated cells. Interestingly, ATP synthase of complex V appears as dimers in the cristae and are responsible for its folding and curvature and thus for the large increase in available surface, that makes mitochondria efficient in energy production (Strauss et al., 2008). This may also contribute to the larger quantity of organelles with multi-folded, concentric cristae in fibroblasts in response to 3O-C_12_-HSL (Figure 3E).

The bioenergetic status of the mitochondria can furthermore regulate organelle signaling and immune function during infection and inflammation. Thus, complexes I and II are the major sites of the production of mitochondrial reactive oxygen species (ROS) and pro-inflammatory cytokine IL-1β during bacterial infection, but the exact mechanisms are still matter of investigation (Mills et al., 2016; Scialo et al., 2017). Indeed, 3O-C_12_-HSL may cause generation of mitochondrial ROS via the calcium uniporter in neutrophils (Singh et al., 2019), and it appears to upregulate the expression of IL-1β (Smith et al., 2002), increase the IL-8 production in epithelial cells and fibroblasts, allowing infiltration of leukocytes into the tissues (Smith et al., 2001), and downregulate the production of yet another pro-inflammatory TNF-α (Telford et al., 1998) and anti-inflammatory IL-10 (Glucksam-Galnoy et al., 2013) in macrophages. Signaling via 3O-C_12_-HSL modulated phagocytosis rate by macrophages (Holm et al., 2015) but this was not accompanied by an increased production of ROS (Vikstrom et al., 2005). These effects may help bacteria survive intracellularly in phagocytes and further colonize and destroy host cells and tissues resulting in a more severe outcome of an infection and inflammation. The role of complex III during bacterial infections has been investigated very little. However, it was shown that 3O-C_12_-HSL can trigger release of mitochondrial cytochrome c via pro-apoptotic pathways in fibroblasts (Schwarzer et al., 2014; Maurice et al., 2019). Thus, there are multidimensional arrays of interactions in infection and inflammation events, where mitochondria take an important role in bioenergetic, signaling and immune status of host cells, and where QS-communication appears to take an impact in pathogenesis of bacteria.

To summarize, our study demonstrates that 3O-C_12_-HSL impacted in a sophisticated and multifactorial way on mitochondrial dynamics and architecture, and thereby their respiration and energetic characteristics. Moreover, this was substantiated by a perturbed expression of an array of components in mitochondrial proteome associated with different functional groups. Targeting mitochondria, 3O-C_12_-HSL provokes distinct cell-type specific changes. Thus, in fibroblast, 3O-C_12_-HSL disturbed mitochondrial dynamics and inner membrane ultrastructure, which was supported by dysregulated expression of mitochondrial proteins mostly involved in structural organization. In epithelial cells, 3O-C_12_-HSL perturbed mitochondria by decreasing their respiration and energetics, which mechanistically was accompanied by differentially abundant proteins of respiratory chain complexes and lipid metabolism (Figure 9). Epithelial cells are positioned strategically to provide both physical and immune barriers to pathogens and other environmental agents (Ivanov et al., 2010) and may therefore display different behavior in terms targeting mitochondrial networks in comparison to underlying fibroblasts playing critical roles in immune regulation and supporting connective tissue. Our study provides a foundation for future investigations on mechanistic insights into a dialog between mitochondria and bacteria based on QS communication, and hereby the biology of infection and inflammation.

## Data Availability Statement

The raw data supporting the conclusions of this manuscript will be made available by the authors, without undue reservation, to any qualified researcher.

## Author Contributions

HJ planned, carried out and analyzed proteome experiments with MT as supervisor and EV as co-supervisor. SL and CS contributed with expertise and resources for Seahorse experiments. MN carried out TEM experiments. EV, MT, MN, and CS contributed to planning and performing experiments, data analysis and interpretation of the results. EV is PI of the project and designed the study, planned and did experiments, evaluated results, drafted and finalized the manuscript. All authors edited and approved the final version of the manuscript.

## Conflict of Interest

The authors declare that the research was conducted in the absence of any commercial or financial relationships that could be construed as a potential conflict of interest.

## Footnotes

1 https://www.agilent.com/en/solutions/cell-metabolism/mitochondrial-disease-research

2 http://www.uniprot.org

3 http://www.ncbi.nlm.nih.gov/pubmed/18940858

## Supplementary Material

FIGURE S1 Cell viability in 3O-C_12_-HSL-treated cells. Two cell types, **(A)** fibroblasts and **(B)** intestinal epithelial cells were either treated with 0.02% DMSO as a diluent (Control), or exposed to 3, 10, 50, or 100 μM 3O-C_12_-HSL for 4, 24, 48, or 72 h, and then analyzed using AlamarBlue assay. Columns represent the mean ± SE. No significant differences are indicated by with “ns,” as analyzed by two-tailed Student’s *t*-test. Data are from at least six independent experiments done on separate days on different cell passages.

TABLE S1 Differentially expressed proteins in mitochondria enriched fraction of fibroblasts after treatment with 10 or 50 μM 3O-C_12_-HSL for 4 h compared to the diluent control.

TABLE S2 Differentially expressed proteins in mitochondria enriched fraction of Caco-2 cells after treatment with 10 or 50 μM 3O-C_12_-HSL for 4 h compared to the diluent control.

TABLE S3 Differentially expressed proteins in mitochondria enriched fraction of fibroblasts after treatment with 10 μM 3O-C_12_-HSL for 4 h compared to the diluent control, Student’s *t*-test.

TABLE S4 Differentially expressed proteins in mitochondria enriched fraction of fibroblasts after treatment with 50 μM 3O-C_12_-HSL for 4 h compared to the diluent control, Student’s *t*-test.

TABLE S5 Differentially expressed proteins in mitochondria enriched fraction of Caco-2 cells treated with 10 μM C12-HSL for 4 h compared to the diluent control, Student’s *t*-test.

TABLE S6 Differentially expressed proteins in mitochondria enriched fraction of Caco-2 cells treated with 50 μM C12-HSL for 4 h compared to the diluent control, Student’s *t*-test.

## Abbreviations

3O-C_12_-HSL: *N*-3-oxo-dodecanoyl-L-homoserine lactone
C_4_-HSL: *N*-butyryl -L-homoserine lactone
AHL: *N*-acylhomoserine lactone
ATP: adenosine triphosphate
DMEM: Dulbecco’s modified Eagle’s medium
DMSO: dimethylsulfoxide
DTT: dithiothreitol
ETC: electron transport chain
FCCP: carbonyl cyanide 4-(trifluoromethoxy) phenylhydrazone
IAA: iodoacetamide
LC-MS/MS: liquid chromatography tandem mass spectrometry
MICOS: mitochondrial contact site and cristae organizing system
MMP: mitochondrial membrane potential
OCR: oxygen consumption rate
PBS: phosphate buffered saline
QS: quorum sensing
RT: room temperature
TEM: transmission electron microscopy.
