# Supplementary figures and images for: Pseudomonas aeruginosa N-3-Oxo-Dodecanoyl-Homoserine Lactone Impacts Mitochondrial Networks Morphology, Energetics, and Proteome in Host Cells
Source: Front Microbiol. 2020 May 25;11:1069. doi: 10.3389/fmicb.2020.01069 (PMC7261938; doi:10.3389/fmicb.2020.01069)

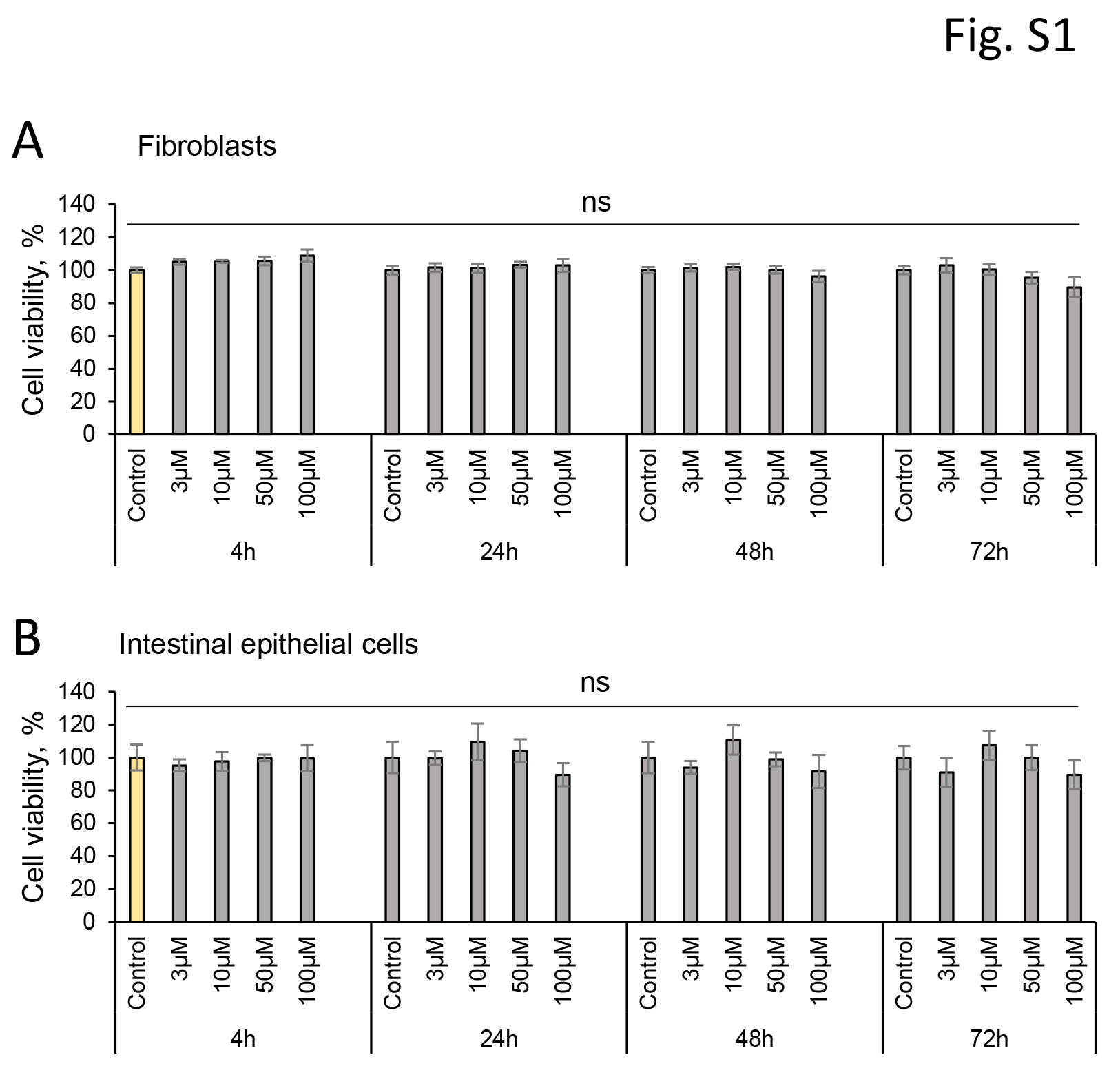

Supplement: FIGURE S1 — Cell viability in 3O-C12-HSL-treated cells. Two cell types, (A) fibroblasts and (B) intestinal epithelial cells were either treated with 0.02% DMSO as a diluent (Control), or exposed to 3, 10, 50, or 100 μM 3O-C12-HSL for 4, 24, 48, or 72 h, and then analyzed using AlamarBlue assay. Columns represent the mean ± SE. No significant differences are indicated by with “ns,” as analyzed by two-tailed Student’s t-test. Data are from at least six independent experiments done on separate days on different cell passages. [file Image_1.TIF]
