# Supplementary material for: Pseudomonas aeruginosa N-3-Oxo-Dodecanoyl-Homoserine Lactone Impacts Mitochondrial Networks Morphology, Energetics, and Proteome in Host Cells
Source: Front Microbiol. 2020 May 25;11:1069. doi: 10.3389/fmicb.2020.01069 (PMC7261938; doi:10.3389/fmicb.2020.01069)
Supplement: TABLE S1 — Differentially expressed proteins in mitochondria enriched fraction of fibroblasts after treatment with 10 or 50 μM 3O-C12-HSL for 4 h compared to the diluent control. [file Data_Sheet_2.zip › Table S1.docx]

**Table S1.** Differentially expressed proteins in mitochondria enriched fraction of fibroblasts after treatment with 10 or 50 µM 3O-C_12_-HSL for 4 h compared to the diluent control.

| Identified proteins | Uniprot accession | MW  kDa | *P-*value  emPAI ANOVA | Quantitative profile | | |
| --- | --- | --- | --- | --- | --- | --- |
|  |  |  |  | Control | 10 µM | 50 µM |
| DnaJ homolog subfamily A member 1 | DNJA1_MOUSE | 45 | 0.012 | high | high | low |
| Nascent polypeptide-associated complex subunit alpha, muscle-specific form | NACAM_MOUSE | 220 | 0.0035 | high | low | high |
| Cluster of Triosephosphate isomerase | TPIS_MOUSE | 32 | 0.005 | high | low | high |
| Cluster of ADP/ATP translocase 2 | ADT2_MOUSE | 33 | 0.011 | high | low | high |
| EH domain-containing protein 4 | EHD4_MOUSE | 61 | 0.015 | high | low | high |
| Membrane-associated progesterone receptor component 2 | PGRC2_MOUSE | 23 | 0.044 | high | low | high |
| Prohibitin | PHB_MOUSE | 30 | 0.0043 | low | high | low |
| Metaxin-2 | MTX2_MOUSE | 30 | 0.024 | low | high | low |
| 39S ribosomal protein L38, mitochondrial | RM38_MOUSE | 45 | 0.05 | low | high | low |
| Isoform 2 of Regulator of nonsense transcripts 1 | RENT1_MOUSE | 123 | 0.0072 | low | low | high |
| Src substrate cortactin | SRC8_MOUSE | 61 | 0.021 | low | low | high |
| 40S ribosomal protein S28 | G3UYV7_MOUSE | 6 | 0.037 | low | low | high |
| Cluster of Ras-related protein Rab-14 | RAB14_MOUSE | 24 | 0.046 | low | low | high |
| Thioredoxin-dependent peroxide reductase, mitochondrial | PRDX3_MOUSE | 28 | 0.042 | high | low | low |
