# Supplementary material for: Pseudomonas aeruginosa N-3-Oxo-Dodecanoyl-Homoserine Lactone Impacts Mitochondrial Networks Morphology, Energetics, and Proteome in Host Cells
Source: Front Microbiol. 2020 May 25;11:1069. doi: 10.3389/fmicb.2020.01069 (PMC7261938; doi:10.3389/fmicb.2020.01069)
Supplement: TABLE S1 — Differentially expressed proteins in mitochondria enriched fraction of fibroblasts after treatment with 10 or 50 μM 3O-C12-HSL for 4 h compared to the diluent control. [file Data_Sheet_2.zip › Table S2.docx]

**Table S2.** Differentially expressed proteins in mitochondria enriched fraction of Caco-2 cells after treatment with 10 or 50 µM 3O-C_12_-HSL for 4 h compared to the diluent control.

| Identified proteins | Uniprot accession | MW  kDa | *P*-value  emPAI ANOVA | Quantitative profile | | |
| --- | --- | --- | --- | --- | --- | --- |
|  |  |  |  | Control | 10 µM | 50 µM |
| Peroxiredoxin-5, mitochondrial | PRDX5_HUMAN | 22 | 0.0024 | low | high | high |
| Aspartyl/asparaginyl beta-hydroxylase | ASPH_HUMAN | 86 | 0.0097 | low | high | high |
| NADH dehydrogenase [ubiquinone] 1 alpha subcomplex subunit 8 | NDUA8_HUMAN | 20 | 0.011 | low | high | high |
| 60S ribosomal protein L5 | RL5_HUMAN | 34 | 0.013 | low | high | high |
| Periplakin | K7EKI8_HUMAN | 204 | 0.021 | low | high | high |
| Prostaglandin E synthase 2 | PGES2_HUMAN | 42 | 0.039 | low | high | high |
| Carboxypeptidase D | CBPD_HUMAN | 153 | 0.05 | low | high | high |
| Cluster of Utrophin | UTRO_HUMAN | 394 | 0.002 | high | high | low |
| ADP-ribosylation factor 6 | ARF6_HUMAN | 20 | 0.0021 | high | high | low |
| Junctional adhesion molecule A | JAM1_HUMAN | 33 | 0.0044 | high | high | low |
| Isoform 2 of Niban-like protein 1 | NIBL1_HUMAN | 83 | 0.005 | high | high | low |
| Cluster of Cytoplasmic FMR1-interacting protein 1 | CYFP1_HUMAN | 145 | 0.0053 | high | high | low |
| Ras GTPase-activating-like protein IQGAP1 | IQGA1_HUMAN | 189 | 0.0054 | high | high | low |
| Cluster of Tight junction protein 1 | G3V1L9_HUMAN | 197 | 0.0093 | high | high | low |
| Phosphatidylethanolamine-binding protein 1 | PEBP1_HUMAN | 21 | 0.023 | high | high | low |
| Beta-2-microglobulin | B2MG_HUMAN | 14 | 0.027 | high | high | low |
| Peptidyl-prolyl cis-trans isomerase FKBP2 | FKBP2_HUMAN | 16 | 0.027 | high | high | low |
| CAD protein | F8VPD4_HUMAN | 236 | 0.028 | high | high | low |
| Cluster of Disks large homolog 1 | A0A0C4DFT3_HUMAN | 100 | 0.029 | high | high | low |
| Brain-specific angiogenesis inhibitor 1-associated protein 2 | I3L4C2_HUMAN | 61 | 0.031 | high | high | low |
| Cluster of LIM domain and actin-binding protein 1 | LIMA1_HUMAN | 85 | 0.031 | high | high | low |
| EH domain-containing protein 4 | EHD4_HUMAN | 61 | 0.036 | high | high | low |
| Isoform 2 of 39S ribosomal protein L47, mitochondrial | RM47_HUMAN | 27 | 0.041 | high | high | low |
| Isoform 1 of Vinculin | VINC_HUMAN | 117 | 0.044 | high | high | low |
| Peroxisomal bifunctional enzyme | ECHP_HUMAN | 79 | 0.02 | high | low | high |
| Isoform 3 of Importin-5 | IPO5_HUMAN | 126 | 0.036 | high | low | high |
| Non-histone chromosomal protein HMG-14 | HMGN1_HUMAN | 11 | 0.011 | high | low | high |
| Isoform 3 of Ubiquitin carboxyl-terminal hydrolase 7 | UBP7_HUMAN | 126 | 0.028 | high | low | high |
| Cluster of Elongation factor 1-alpha 1 | EF1A1_HUMAN | 50 | 0.035 | high | low | high |
| Cytochrome c1, heme protein, mitochondrial | CY1_HUMAN | 35 | 0.04 | high | low | high |
| Heterochromatin protein 1-binding protein 3 | HP1B3_HUMAN | 61 | 0.04 | high | low | high |
| Isoform 2 of Oxysterol-binding protein-related protein 8 | OSBL8_HUMAN | 100 | 0.0069 | low | low | high |
| Cluster of Isoform 3 of Heterogeneous nuclear ribonucleoprotein A/B | ROAA_HUMAN | 31 | 0.011 | low | low | high |
| Cluster of Trifunctional enzyme subunit alpha, mitochondrial | ECHA_HUMAN | 83 | 0.015 | low | low | high |
| ATP-dependent RNA helicase A | DHX9_HUMAN | 141 | 0.021 | low | low | high |
| Insulin-like growth factor 2 mRNA-binding protein 2 | F8W930_HUMAN | 67 | 0.022 | low | low | high |
| Cluster of NAD | NNTM_HUMAN | 114 | 0.024 | low | low | high |
| Adipocyte plasma membrane-associated protein | H0Y512_HUMAN | 45 | 0.026 | low | low | high |
| Cluster of Diacylglycerol O-acyltransferase 1 | DGAT1_HUMAN | 55 | 0.027 | low | low | high |
| GrpE protein homolog 1, mitochondrial | GRPE1_HUMAN | 24 | 0.034 | low | low | high |
| Cluster of Isoform 2 of MICOS complex subunit MIC60 | MIC60_HUMAN | 83 | 0.035 | low | low | high |
| Stress-70 protein, mitochondrial | GRP75_HUMAN | 74 | 0.041 | low | low | high |
| Isoform B of Manganese-transporting ATPase 13A1 | AT131_HUMAN | 121 | 0.042 | low | low | high |
| Isoform 2 of Triosephosphate isomerase | TPIS_HUMAN | 27 | 0.043 | low | low | high |
| Heterogeneous nuclear ribonucleoprotein H3 | HNRH3_HUMAN | 37 | 0.045 | low | low | high |
| Cluster of HLA class I histocompatibility antigen, A-2 alpha chain | 1A02_HUMAN | 41 | 0.045 | low | low | high |
| Cluster of Prenylcysteine oxidase 1 | PCYOX_HUMAN | 57 | 0.046 | low | low | high |
| Cluster of Alpha-actinin-1 | ACTN1_HUMAN | 103 | 0.0021 | high | low | low |
| 60S ribosomal protein L15 | RL15_HUMAN | 24 | 0.0074 | high | low | low |
| Succinate--CoA ligase [ADP/GDP-forming] subunit alpha, mitochondrial | SUCA_HUMAN | 36 | 0.0078 | high | low | low |
| Succinate dehydrogenase assembly factor 2, mitochondrial | F5GYJ5_HUMAN | 18 | 0.0091 | high | low | low |
| RNA cytidine acetyltransferase | A0A087WV29_HUMAN | 94 | 0.01 | high | low | low |
| Cytochrome c oxidase subunit 5B, mitochondrial | COX5B_HUMAN | 14 | 0.012 | high | low | low |
| Cluster of Filamin-A | FLNA_HUMAN | 281 | 0.016 | high | low | low |
| Cluster of Isoform 2 of 3-keto-steroid reductase | DHB7_HUMAN | 37 | 0.022 | high | low | low |
| 40S ribosomal protein S25 | RS25_HUMAN | 14 | 0.028 | high | low | low |
| TAR DNA-binding protein 43 | A0A087X260_HUMAN | 34 | 0.037 | high | low | low |
| Cluster of Actin-related protein 3 | ARP3_HUMAN | 47 | 0.047 | high | low | low |
| Ubiquitin thioesterase OTUB1 | F5GYJ8_HUMAN | 32 | 0.047 | high | low | low |
