# Supplementary material for: Pseudomonas aeruginosa N-3-Oxo-Dodecanoyl-Homoserine Lactone Impacts Mitochondrial Networks Morphology, Energetics, and Proteome in Host Cells
Source: Front Microbiol. 2020 May 25;11:1069. doi: 10.3389/fmicb.2020.01069 (PMC7261938; doi:10.3389/fmicb.2020.01069)
Supplement: TABLE S1 — Differentially expressed proteins in mitochondria enriched fraction of fibroblasts after treatment with 10 or 50 μM 3O-C12-HSL for 4 h compared to the diluent control. [file Data_Sheet_2.zip › Table S3.docx]

**Table S3.** Differentially expressed proteins in mitochondria enriched fraction of fibroblasts after treatment with 10 µM 3O-C_12_-HSL for 4 h compared to the diluent control, Students *t*-test.

| Protein | Gene | *P*-value  emPAI | *P*-value  NSAF | Fold change  emPAI | Fold change  NSAF |
| --- | --- | --- | --- | --- | --- |
| 39S ribosomal protein L38, mitochondrial | Mrpl38 | 0.02 | 0.018 | 7.6 | 6.8 |
| Small nuclear ribonucleoprotein Sm D2 | Snrpd2 | 0.049 |  | 7.1 |  |
| Metaxin-2 | Mtx2 | 0.039 | 0.04 | 6.6 | 6 |
| Valine--tRNA ligase (Fragment) | Vars | 0.043 | 0.037 | 3.2 | 3.2 |
| ERO1-like protein alpha | Ero1a | 0.017 | 0.016 | 1.6 | 1.6 |
| Prohibitin | Phb | 0.0076 |  | 1.5 |  |
| Src substrate cortactin | Cttn | 0.05 |  | 0.8 |  |
| Cluster of ADP/ATP translocase 2 | Slc25a5 | 0.046 |  | 0.7 |  |
| Thioredoxin-dependent peroxide reductase, mitochondrial | Prdx3 | 0.005 | 0.006 | 0.6 | 0.7 |
| Galectin-1 | Lgals1 | 0.043 |  | 0.6 |  |
| Histone H2A.V | H2afv | 0.047 |  | 0.6 |  |
| Acetolactate synthase-like protein | Ilvbl | 0.017 |  | 0.6 |  |
| Nascent polypeptide-associated complex subunit alpha, muscle-specific form | Naca | 0.0063 | 0.0096 | 0.5 | 0.5 |
| EH domain-containing protein 4 | Ehd4 | 0.011 | 0.014 | 0.4 | 0.5 |
| ATP-dependent RNA helicase DDX1 | Ddx1 | 0.026 | 0.035 | 0.4 | 0.4 |
| Isoform 2 of Poly(U)-binding-splicing factor PUF60 | Puf60 | 0.026 | 0.022 | 0.2 | 0.2 |
| Cluster of Triosephosphate isomerase | Tpi1 | 0.016 |  | 0 |  |
