# Supplementary material for: Pseudomonas aeruginosa N-3-Oxo-Dodecanoyl-Homoserine Lactone Impacts Mitochondrial Networks Morphology, Energetics, and Proteome in Host Cells
Source: Front Microbiol. 2020 May 25;11:1069. doi: 10.3389/fmicb.2020.01069 (PMC7261938; doi:10.3389/fmicb.2020.01069)
Supplement: TABLE S1 — Differentially expressed proteins in mitochondria enriched fraction of fibroblasts after treatment with 10 or 50 μM 3O-C12-HSL for 4 h compared to the diluent control. [file Data_Sheet_2.zip › Table S4.docx]

**Table S4.** Differentially expressed proteins in mitochondria enriched fraction of fibroblasts after treatment with 50 µM 3O-C_12_-HSL for 4 h compared to the diluent control, Students *t*-test.

| Protein | Gene | *P*-value  emPAI | *P*-value  NSAF | Fold change  emPAI | Fold change  NSAF |
| --- | --- | --- | --- | --- | --- |
| Isoform 2 of Regulator of nonsense transcripts 1 | Upf1 | 0.0078 | 0.0065 | 7.9 | 8.4 |
| Synaptosomal-associated protein | Snap23 | 0.03 | 0.027 | 3.9 | 3.9 |
| Cluster of Eukaryotic translation initiation factor 2A | Eif2a | 0.047 |  | 3.6 |  |
| Cluster of Isoform PLEC-1A of Plectin | Plec |  | 0.035 |  | 3.4 |
| 40S ribosomal protein S28 | Rps28 | 0.015 |  | 2 |  |
| Cluster of Mitochondrial carrier homolog 2 | Mtch2 |  | 0.036 |  | 2 |
| Cluster of Aspartyl/asparaginyl beta-hydroxylase | Asph |  | 0.04 |  | 1.8 |
| ATP synthase F(0) complex subunit B1, mitochondrial | Atp5f1 |  | 0.048 |  | 1.8 |
| Calcium uptake protein 2, mitochondrial | Micu2 | 0.034 | 0.036 | 1.5 | 1.5 |
| Cluster of C-type mannose receptor 2 | Mrc2 | 0.019 |  | 1.4 |  |
| T-complex protein 1 subunit epsilon | Cct5 | 0.018 |  | 1.4 |  |
| Cluster of 60S ribosomal protein L3 | Rpl3 | 0.042 |  | 1.3 |  |
| Sideroflexin-1 | Sfxn1 | 0.021 |  | 0.7 |  |
| T-complex protein 1 subunit delta | Cct4 | 0.049 | 0.039 | 0.6 | 0.5 |
| Lysosomal alpha-glucosidase | Gaa |  | 0.023 |  | 0.6 |
| Probable ATP-dependent RNA helicase DDX5 | Ddx5 |  | 0.042 |  | 0.6 |
| 40S ribosomal protein S17 | Rps17 | 0.013 | 0.02 | 0.4 | 0.5 |
| WD repeat-containing protein 1 | Wdr1 |  | 0.047 |  | 0.4 |
| B-cell receptor-associated protein 31 | Bcap31 | 0.049 | 0.047 | 0.3 | 0.3 |
| DnaJ homolog subfamily A member 1 | Dnaja1 | 0.01 | 0.0079 | 0.2 | 0.2 |
| Disabled homolog 2 | Dab2 | 0.045 |  | 0.1 |  |
| Transmembrane protein 43 | Tmem43 | 0.03 | 0.028 | 0 | 0 |
