# Supplementary material for: Pseudomonas aeruginosa N-3-Oxo-Dodecanoyl-Homoserine Lactone Impacts Mitochondrial Networks Morphology, Energetics, and Proteome in Host Cells
Source: Front Microbiol. 2020 May 25;11:1069. doi: 10.3389/fmicb.2020.01069 (PMC7261938; doi:10.3389/fmicb.2020.01069)
Supplement: TABLE S1 — Differentially expressed proteins in mitochondria enriched fraction of fibroblasts after treatment with 10 or 50 μM 3O-C12-HSL for 4 h compared to the diluent control. [file Data_Sheet_2.zip › Table S5.docx]

**Table S5.** Differentially expressed proteins in mitochondria enriched fraction of Caco-2 cells treated with 10 µM C12-HSL for 4 h compared to the diluent control, Students *t*-test.

| Protein | Gene | *P*-value  emPAI | *P*-value  NSAF | Fold change emPAI | Fold change NSAF |
| --- | --- | --- | --- | --- | --- |
| Non-histone chromosomal protein HMG-14 | HMGN1 | 0.032 | 0.026 | INF | INF |
| Isoform 3 of Ubiquitin carboxyl-terminal hydrolase 7 | UBP7 | 0.033 | 0.026 | 6 | 6 |
| Cluster of E3 SUMO-protein ligase RanBP2 | RBP2 | 0.0031 | 0.0057 | 3.9 | 3.2 |
| Cluster of AT-rich interactive domain-containing protein 3A | ARI3A | 0.041 |  | 3 |  |
| Heterochromatin protein 1-binding protein 3 | HP1B3 | 0.018 | 0.019 | 2.6 | 3 |
| NADH dehydrogenase | NDUA8 | 0.018 | 0.018 | 2.4 | 2.3 |
| 60S ribosomal protein L23a | A8MUS3 | 0.013 | 0.016 | 2.1 | 1.9 |
| Protein S100-A11 | S10AB | 0.032 | 0.034 | 2.1 | 1.8 |
| 60S ribosomal protein L5 | RL5 | 0.013 | 0.019 | 1.8 | 1.7 |
| Prostaglandin E synthase 2 | PGES2 | 0.027 | 0.02 | 1.8 | 1.7 |
| Cluster of Isoform 4 of Alpha-methylacyl-CoA racemase | AMACR | 0.027 | 0.041 | 1.7 | 1.7 |
| Periplakin | K7EKI8 | 0.0056 | 0.0023 | 1.6 | 1.6 |
| Superoxide dismutase | SODC | 0.024 | 0.028 | 1.6 | 1.5 |
| 60S ribosomal protein L8 | RL8 | 0.035 | 0.031 | 1.6 | 1.4 |
| Aspartyl/asparaginyl beta-hydroxylase | ASPH | 0.021 | 0.024 | 1.5 | 1.4 |
| Cluster of Prosaposin | SAP |  | 0.042 |  | 1.5 |
| Isoform 2 of Transgelin-2 | TAGL2 | 0.011 |  | 1.4 |  |
| Peroxiredoxin-5, mitochondrial | PRDX5 | 0.018 |  | 1.4 |  |
| Cytochrome c1, heme protein, mitochondrial | CY1 |  | 0.018 |  | 1.4 |
| Cluster of Elongation factor 1-alpha 1 | EF1A1 | 0.013 |  | 1.3 |  |
| Heterogeneous nuclear ribonucleoprotein A0 | ROA0 | 0.044 |  | 1.3 |  |
| Cluster of 3-ketoacyl-CoA thiolase, mitochondrial | THIM |  | 0.033 |  | 1.3 |
| Cluster of Isoform 2 of Keratin, type II cytoskeletal 8 | K2C8 |  | 0.042 |  | 1.3 |
| Isoform 2 of Transgelin-2 | TAGL2 |  | 0.04 |  | 1.2 |
| Cluster of Alpha-actinin-1 | ACTN1 | 0.024 | 0.014 | 0.8 | 0.8 |
| Dihydrolipoyllysine-residue acetyltransferase component of pyruvate dehydrogenase complex, mitochondrial | ODP2 |  | 0.047 |  | 0.8 |
| Isoform 2 of Myosin-14 | MYH14 | 0.026 |  | 0.7 |  |
| Cluster of Electron transfer flavoprotein subunit beta | ETFB | 0.049 |  | 0.7 |  |
| 60S ribosomal protein L15 | RL15 | 0.024 | 0.024 | 0.6 | 0.6 |
| Peroxisomal bifunctional enzyme | ECHP | 0.025 | 0.022 | 0.6 | 0.6 |
| Cytochrome c oxidase subunit 5B, mitochondrial | COX5B | 0.027 | 0.027 | 0.6 | 0.6 |
| Pyruvate dehydrogenase phosphatase regulatory subunit, mitochondrial | PDPR | 0.043 | 0.04 | 0.5 | 0.5 |
| Isoform 2 of Plectin | PLEC | 0.046 |  | 0.5 |  |
| Protein dpy-30 homolog | DPY30 |  | 0.04 |  | 0.5 |
| Cluster of Ephrin type-B receptor 2 | B1AKC9 |  | 0.042 |  | 0.5 |
| 40S ribosomal protein S25 | RS25 |  | 0.044 |  | 0.5 |
| Cluster of Isoform 2 of Ras-related protein Ral-B | RALB | 0.012 | 0.025 | 0.4 | 0.4 |
| Isoform 2 of Nck-associated protein 1 | NCKP1 |  | 0.049 |  | 0.4 |
| m-AAA protease-interacting protein 1, mitochondrial | MAIP1 | 0.045 | 0.045 | 0.3 | 0.3 |
| Succinate dehydrogenase assembly factor 2, mitochondrial | F5GYJ5 | 0.0073 | 0.0039 | 0.2 | 0.2 |
| RNA cytidine acetyltransferase | A0A087WV29 | 0.021 | 0.021 | 0.2 | 0.2 |
| Sigma intracellular receptor 2 | SGMR2 | 0.048 | 0.05 | 0.2 | 0.2 |
| Cluster of Isoform 2 of Voltage-dependent anion-selective channel protein 3 | VDAC3 |  | 0.033 |  | 0.2 |
| Cluster of Isoform 2 of 3-keto-steroid reductase | DHB7 | 0.05 | 0.05 | 0 | 0 |
