# Supplementary material for: Pseudomonas aeruginosa N-3-Oxo-Dodecanoyl-Homoserine Lactone Impacts Mitochondrial Networks Morphology, Energetics, and Proteome in Host Cells
Source: Front Microbiol. 2020 May 25;11:1069. doi: 10.3389/fmicb.2020.01069 (PMC7261938; doi:10.3389/fmicb.2020.01069)
Supplement: TABLE S1 — Differentially expressed proteins in mitochondria enriched fraction of fibroblasts after treatment with 10 or 50 μM 3O-C12-HSL for 4 h compared to the diluent control. [file Data_Sheet_2.zip › Table S6.docx]

**Table S6.** Differentially expressed proteins in mitochondria enriched fraction of Caco-2 cells treated with 50 µM C12-HSL for 4 h compared to the diluent control, Students *t*-test.

| Protein | Gene | *P*-value  emPAI | *P*-value  NSAF | Fold change  emPAI | Fold change  NSAF |
| --- | --- | --- | --- | --- | --- |
| Cluster of Erlin-2 | ERLIN2 |  | 0.022 |  | INF |
| Isoform 2 of Oxysterol-binding protein-related protein 8 | OSBPL8 | 0.032 | 0.033 | 9 | 8 |
| Isoform 4 of CDK5 regulatory subunit-associated protein 3 | CDK5RAP3 | 0.047 |  | 6.3 |  |
| Transmembrane protein 59 | TMEM59 | 0.017 | 0.018 | 6.1 | 5.4 |
| Cluster of Diacylglycerol O-acyltransferase 1 | DGAT1 | 0.04 |  | 4.2 |  |
| Cluster of NAD | NNT | 0.0072 | 0.031 | 2.9 | 5.5 |
| NADH dehydrogenase | NDUFA8 | 0.0068 | 0.0075 | 2.5 | 2.3 |
| Cluster of Isoform 3 of Heterogeneous nuclear ribonucleoprotein A/B | HNRNPAB | 0.013 | 0.033 | 2.4 | 2.1 |
| Adipocyte plasma membrane-associated protein | APMAP | 0.0052 | 0.013 | 2.3 | 2.1 |
| Heterogeneous nuclear ribonucleoprotein H3 | HNRNPH3 | 0.018 | 0.017 | 2.2 | 2 |
| 60S ribosomal protein L5 | RPL5 | 0.0093 | 0.012 | 2.1 | 1.8 |
| Cluster of Heterogeneous nuclear ribonucleoproteins C1/C2 | HNRNPC | 0.038 |  | 2.1 |  |
| Golgi integral membrane protein 4 | GOLIM4 | 0.021 | 0.018 | 1.9 | 1.8 |
| 10 kDa heat shock protein, mitochondrial | HSPE1 | 0.027 |  | 1.9 |  |
| Polypeptide N-acetylgalactosaminyltransferase 6 | GALNT6 | 0.029 |  | 1.9 |  |
| Inositol 1,4,5-trisphosphate receptor type 3 | ITPR3 | 0.047 | 0.049 | 1.9 | 1.8 |
| Reticulocalbin-1 | RCN1 |  | 0.023 |  | 1.9 |
| Aspartyl/asparaginyl beta-hydroxylase | ASPH | 0.0082 | 0.0072 | 1.8 | 1.7 |
| Peroxiredoxin-5, mitochondrial | PRDX5 | 0.0032 | 0.012 | 1.7 | 1.4 |
| Cluster of Prenylcysteine oxidase 1 | PCYOX1 | 0.012 | 0.0028 | 1.7 | 1.5 |
| Prostaglandin E synthase 2 | PTGES2 | 0.019 | 0.017 | 1.7 | 1.6 |
| Polypeptide N-acetylgalactosaminyltransferase 6 | GALNT6 | 0.015 |  | 1.7 |  |
| Insulin-like growth factor 2 mRNA-binding protein 2 | IGF2BP2 | 0.0088 | 0.041 | 1.6 | 1.6 |
| Isoform 2 of 3-mercaptopyruvate sulfurtransferase | MPST | 0.021 | 0.023 | 1.6 | 1.5 |
| Heterogeneous nuclear ribonucleoproteins A2/B1 | HNRNPA2B1 | 0.038 |  | 1.6 |  |
| Heterogeneous nuclear ribonucleoprotein F | HNRNPF |  | 0.037 |  | 1.6 |
| Carboxypeptidase D | CPD | 0.0076 | 0.0092 | 1.5 | 1.4 |
| Cluster of Isoform 2 of Pyruvate dehydrogenase E1 component subunit beta, mitochondrial | PDHB | 0.034 |  | 1.5 |  |
| Cluster of Trifunctional enzyme subunit alpha, mitochondrial | HADHA | 0.012 | 0.0037 | 1.4 | 1.3 |
| Cluster of Reticulon-4 | RTN4 | 0.024 |  | 1.4 |  |
| ATP-dependent RNA helicase A | DHX9 | 0.026 | 0.037 | 1.4 | 1.3 |
| Cluster of Glucosidase 2 subunit beta | PRKCSH | 0.017 | 0.032 | 1.3 | 1.2 |
| Isoform 2 of Triosephosphate isomerase | TPI1 | 0.026 | 0.018 | 1.3 | 1.2 |
| Endoplasmic reticulum resident protein 44 | ERP44 | 0.043 |  | 1.3 |  |
| Cluster of 60S acidic ribosomal protein P2 | RPLP2 |  | 0.024 |  | 1.3 |
| X-ray repair cross-complementing protein 5 | XRCC5 |  | 0.024 |  | 1.3 |
| 60S ribosomal protein L4 | RPL4 |  | 0.026 |  | 1.3 |
| Stress-70 protein, mitochondrial | HSPA9 | 0.031 |  | 1.2 |  |
| Cluster of Isoform 2 of Annexin A2 | ANXA2 |  | 0.04 |  | 1.2 |
| Cluster of Isoform 2 of Keratin, type II cytoskeletal 8 | KRT8 |  | 0.048 |  | 1.2 |
| Cluster of Isoform 4 of Sodium/potassium-transporting ATPase subunit alpha-1 | ATP1A1 | 0.04 |  | 0.9 |  |
| Cluster of Spectrin alpha chain, non-erythrocytic 1 | SPTAN1 | 0.028 |  | 0.8 |  |
| Cluster of Cadherin-17 | CDH17 | 0.045 |  | 0.8 |  |
| Cluster of Alpha-actinin-1 | ACTN1 | 0.00031 | 0.00072 | 0.7 | 0.7 |
| Cluster of Filamin-A | FLNA | 0.013 | 0.0047 | 0.7 | 0.6 |
| Cluster of Plastin-3 | PLS3 | 0.02 | 0.006 | 0.7 | 0.6 |
| Unconventional myosin-Ic | MYO1C | 0.024 | 0.027 | 0.7 | 0.7 |
| T-complex protein 1 subunit theta | CCT8 | 0.027 | 0.021 | 0.7 | 0.7 |
| Cluster of Actin-related protein 3 | ACTR3 | 0.034 |  | 0.7 |  |
| Cluster of Isoform 3 of Serine hydroxymethyltransferase, mitochondrial | SHMT2 |  | 0.023 |  | 0.7 |
| Isoform 6 of Inactive tyrosine-protein kinase 7 | PTK7 |  | 0.05 |  | 0.7 |
| Ras GTPase-activating-like protein IQGAP1 | IQGAP1 | 0.0013 | 0.0018 | 0.6 | 0.7 |
| Cluster of LIM domain and actin-binding protein 1 | LIMA1 | 0.002 |  | 0.6 |  |
| Isoform 2 of Neural cell adhesion molecule L1 | L1CAM | 0.006 | 0.0051 | 0.6 | 0.6 |
| Protein S100-A16 | S100A16 | 0.021 | 0.028 | 0.6 | 0.7 |
| Guanine nucleotide-binding protein subunit alpha-13 | GNA13 | 0.03 | 0.02 | 0.6 | 0.5 |
| Cluster of Isoform 2 of Fructose-bisphosphate aldolase A | ALDOA |  | 0.0033 |  | 0.6 |
| 60S ribosomal protein L15 | RPL15 |  |  |  |  |
| Talin-1 | TLN1 |  | 0.039 |  | 0.6 |
| EH domain-containing protein 1 | EHD1 |  | 0.044 |  | 0.6 |
| Cluster of Tight junction protein 1 | TJP1 | 0.00065 | 0.0016 | 0.5 | 0.5 |
| Isoform 2 of Niban-like protein 1 | FAM129B | 0.00073 | 0.0048 | 0.5 | 0.6 |
