# Supplementary material for: Pseudomonas aeruginosa N-3-Oxo-Dodecanoyl-Homoserine Lactone Impacts Mitochondrial Networks Morphology, Energetics, and Proteome in Host Cells
Source: Front Microbiol. 2020 May 25;11:1069. doi: 10.3389/fmicb.2020.01069 (PMC7261938; doi:10.3389/fmicb.2020.01069)
Supplement: Supplementary file 3 [file Data_Sheet_1.docx]

**Method S1. Cell viability assay.** Cell viability was analyzed by measuring metabolic activity of cells cultured in 24-well plates using AlamarBlue assay (Life Technologies) according to the manufacturer’s recommendations. After treatment with 3O-C_12_-HSL, cells were rinsed with PBS, pH 7.3 and incubated with DMEM containing 10 % AlamarBlue solution for 2 h at 37°C in 5% CO_2_. Following incubation, 150 µl aliquotes were placed into 96-well plate and analysed in plate reader (VICTOR X4 2030, PerkinElmer) at the excitation wavelength of 550 nm and emission of 610 nm. Calculation of cells metabolic activity of the cells (AlamarBlue reduction) was done by subtracting the fluorescence values of non-reduced blank control samples without cells from the samples containing cells treated with diluent (Control) och 3O-C_12_-HSL at different concentrations. At least 6 independent experiments were done on separate days on different cell passages.
